# The Cerebellar Connectome

**DOI:** 10.1007/s12311-026-02042-x

**Published:** 2026-07-16

**Authors:** Oliver Schmitt, Paulina Morawska, Vishnu Prathapan, Peter Eipert

**Affiliations:** 1 MSH Medical School Hamburg – University of Applied Sciences and Medical University, Hamburg, Germany; 2 University Medical Center Rostock, Rostock, Germany

**Keywords:** Cerebellum, Connectome, Network neuroscience, Rat brain, Graph theory, Dynamic modeling

## Abstract

The cerebellum, long known for its role in motor control, has increasingly been implicated in cognitive and affective functions. Despite this broadened perspective, its connectivity remains undercharacterized relative to the cerebral cortex. Here, we present the first comprehensive cerebellar connectome analysis derived from a large-scale, meta-analytic database of over 7,800 high-resolution tract-tracing studies in the rat brain. Leveraging the neuroVIISAS framework, we constructed a directionally weighted, hierarchically organized cerebellar subnetwork integrating both intrinsic and extrinsic connections, including lateralization and interhemispheric projections. Our methodological pipeline involved region expansion, graph-theoretical filtering, and systematic edge weighting by anatomical significance. The resulting network, encompassing 862 regions and over 21,000 edges, was analyzed across multiple topological scales. Mesoscale analysis revealed hallmark properties of small-world and scale-free networks, while motif and modularity analyses identified non-random, functionally coherent microcircuits and subsystems. Local connectome metrics uncovered key integrative hubs–especially within brainstem-cerebellar loops–and exposed gradients of modularity, controllability, and vulnerability. A novel vulnerability analysis showed that the removal of high-significance edges leads to rapid and irregular degradation of clustering in the empirical network, in contrast to the robustness of rewired surrogate models. This indicates the presence of structurally privileged bottlenecks essential for cerebellar integration. Our results collectively highlight the cerebellum’s dual design: functionally specialized yet structurally efficient, with both local modularity and long-range integration. This study establishes a robust foundation for future multimodal, dynamic, and cross-species connectomic research. Integrating empirical data on neuronal dynamics, synaptic plasticity, and gene expression will be essential to fully realize the translational potential of cerebellar network models in both health and disease.

## Introduction

Understanding the architecture and function of the brain requires detailed knowledge of its structural and functional connectivity–the connectome. In recent years, connectomics has evolved into a powerful interdisciplinary field, employing tools from neuroanatomy, graph theory, and computational neuroscience to decipher the complex connectomes that underlie brain function [1, 2]. While substantial efforts have been devoted to mapping the connectomes of the cortex and whole-brain macro-connectomes, comparatively less attention has been given to the cerebellum [3–5]. This is despite a growing body of evidence indicating that the cerebellum plays critical roles not only in sensorimotor coordination but also in higher cognitive, affective, and autonomic functions [6]. The cerebellar connectome–the totality of the cerebellum’s intrinsic and extrinsic connections–represents a vital and largely underexplored frontier in systems neuroscience.

Laboratory rats (*Rattus norvegicus*) serve as an established model in neuroscience due to their well-characterized neuroanatomy, genetic manipulability, and translational relevance [7–10]. The study of the cerebellar connectome in rats offers several advantages. First, the cerebellum of rats shares fundamental architectural features with that of primates, including laminar organization, modular circuitry (zones and microzones), and conserved afferent and efferent pathways [11–13]. Second, the rat model provides access to high-resolution tract-tracing and electrophysiological data, which are critical for validating connectome-based inferences derived from graph theory and dynamic systems analysis [14–17].

Investigating the cerebellar connectome in rats also offers practical feasibility. Experimental manipulation of neural circuits–such as optogenetics, viral tracing, and pharmacological perturbation–can be applied systematically to examine the causal relationships between structure and function [18–20]. Moreover, given the extensive use of rat models in studies of neurodevelopmental disorders (e.g., autism), neurodegeneration (e.g., spinocerebellar ataxias), and injury recovery (e.g., stroke), a detailed map of cerebellar connectivity will directly support pathophysiological models and therapeutic interventions [21–23].

Studying the *partial* cerebellar connectome using connectome analysis and graph-theoretic tools allows researchers to extract topological and functional insights from incomplete datasets, which is often the case in neuroanatomical studies [21–23]. Graph theory provides a formal framework for describing how regions (nodes) are interconnected via pathways (edges), enabling the identification of hubs, modular substructures, small-world properties, and critical connectors. Even partial reconstructions–e.g., of specific lobules or circuits–can reveal principles of organization such as symmetry, redundancy, and hierarchical processing [24–26].

Dynamic analysis complements static connectomics by simulating or modeling the flow of information across connectomes over time. Tools such as dynamic causal modeling, connectome controllability analysis, and recurrent neural connectomes can help explore how activity propagates across the cerebellar circuit under different conditions. These analyses are crucial for understanding how the cerebellum integrates sensorimotor inputs, coordinates timing, and contributes to forward modeling–a core computational role ascribed to cerebellar circuits [27–29].

Furthermore, dynamic systems analysis can help determine how cerebellar connectomes respond to perturbations, such as lesions or abnormal patterns of input. This has implications for understanding how cerebellar dysfunction manifests in clinical disorders and how compensatory plasticity occurs following injury [30–32].

One important dimension of cerebellar connectomics is laterality. The cerebellum exhibits a degree of hemispheric specialization and inter-hemispheric coordination, which warrants detailed study of left, right, and contralateral connectomes. Functional imaging and lesion studies in humans have shown that the cerebellum interacts with the contralateral cerebral hemisphere, forming closed-loop cortico-cerebellar circuits that are topographically organized [5, 33, 34]. In rats, similar cross-hemispheric connectivity patterns exist, particularly in sensorimotor domains.

Analyzing left-hemispheric, right-hemispheric, and contralateral cerebellar connectivity can elucidate asymmetries in circuit organization and function [35–37]. For example, are certain lobules or microzones more densely connected or serve as central hubs in one hemisphere versus the other? Are contralateral pathways more prominent in motor than in cognitive domains? Addressing such questions will deepen our understanding of cerebellar computation and its integration into broader neural systems.

Moreover, hemispheric analyses may reveal plasticity mechanisms, as in the case of unilateral cerebellar damage where contralateral structures may adaptively reorganize. Detailed lateralized connectomes could also inform rehabilitation strategies in both preclinical and clinical settings [38–40].

The cerebellar connectome comprises both *intrinsic* and *extrinsic* components. The *intrinsic* cerebellar connectome encompasses the internal microcircuitry of the cerebellar cortex and nuclei, including mossy fiber input pathways, granule and Purkinje cell connectivity [41], climbing fiber modulation, interneuronal interactions, and the output architecture of the deep cerebellar nuclei. This connectome is highly structured and modular, exhibiting a fractal-like arrangement of repeated circuit motifs, which enables the cerebellum to perform parallel, distributed processing of input signals [4, 5, 42–44].

Studying intrinsic connectivity is essential for understanding how the cerebellum performs its canonical computations–timing, prediction, and error correction. It also informs theories of cerebellar function based on internal models and recurrent inhibition. Connectome analysis can identify intrinsic modules, bottlenecks, and synchronization patterns within the cerebellar cortex and nuclei [45–49].

In contrast, the *extrinsic* connectome refers to the connections between the cerebellum and other brain regions, including the cerebral cortex, thalamus, brainstem nuclei, and spinal cord. These pathways mediate the flow of sensory input to the cerebellum and the output signals that influence motor, cognitive, and autonomic systems [50–52]. Extrinsic connectivity is necessary to situate the cerebellum within the larger brain connectome, enabling it to modulate behavior and learning [42, 43, 53–55].

A complete model of cerebellar function must integrate both intrinsic and extrinsic connectivity. For instance, understanding how cortical inputs are processed through intrinsic circuits before being relayed back to the cortex via thalamic pathways allows us to model closed-loop cerebellar functions such as predictive motor control. Additionally, extrinsic connectivity may exhibit developmentally dynamic or experience-dependent patterns, which can be studied using longitudinal connectome models.

In sum, mapping the cerebellar connectome–both in part and in whole–represents a crucial step in advancing our knowledge of brain architecture and computation. By focusing on the rat model and leveraging tools from graph theory and dynamic systems analysis, this study aims to contribute to a systems-level understanding of cerebellar organization. Particular emphasis will be placed on hemispheric and contralateral connectivity, as well as on delineating the roles of intrinsic and extrinsic connectomes. This approach holds the promise of bridging the gap between microcircuit-level studies and whole-brain models, ultimately contributing to a unified theory of cerebellar function in health and disease.

## Material and Methods

We performed a retrospective meta-analytic collation of data from over 7,800 published tract-tracing studies, compiling a comprehensive connectome database [56–60]. In addition to connection strength and directionality, metadata such as tracer type, subject sex, injection sites, labeled regions, and experimental context (e.g., sex, annotations, methodology) and study-specific annotations were included to support multifactorial analyses. This dataset forms the basis for a hierarchically organized, weighted, and directed connectome, where brain regions are structured from macroscopic subdivisions down to subnuclei, and connectivity includes directionality and connection density.

For neuroanatomical modeling and simulation, we employed the neuroVIISAS platform, a comprehensive software framework designed to integrate and analyze large-scale tract-tracing data [61–63].

The framework enables bidirectional access between anatomical atlases (e.g., Paxinos and Watson) [64] and experimental data, allowing users to perform both atlas-driven queries and data-based regional mapping. It supports exporting this structured connectivity into simulation-ready formats, facilitating integration with neuronal simulators such as NEST [65–67], NEURON [68–70] and The Virtual Brain (TVB) [71–73]. Quantitative analysis modules embedded in the framework compute key graph-theoretical measures (e.g., centrality, clustering, path length), providing insights into connectome structure and organization.

Visualization tools within neuroVIISAS allow for both two-dimensional and three-dimensional rendering of the connectome, including tracer injection sites, axonal projections, and lesion simulations. Advanced features further include the integration of non-connectivity data such as gene expression and receptor density (in extended versions), and the application of formal ontologies and semantic structures to support reproducible and consistent queries.

neuroVIISAS represents one of the earliest platforms to unify anatomical connectivity data with computational modeling capabilities and has been used in several studies addressing basal ganglia [74], amygdaloid complex [75] as well as brainstem [15] systems neuroscience, multi-scale connectome modeling [76], and comparative connectomics [63].

To focus the connectomic analysis on cerebellar circuitry, a targeted extraction of relevant brain regions was performed. Specifically, regions of interest (ROIs) encompassing the intrinsic substructures of the cerebellum, as well as extrinsic regions with known direct or indirect connectivity, were selected based on hierarchical anatomical ontologies. This included both central and peripheral components of the nervous system as cataloged in the tract-tracing dataset.

The selection was guided by established neuroanatomical knowledge of cerebellar circuitry, ensuring that the resulting network remains interpretable in a biological context. While purely data-driven approaches may capture a broader range of connections, they may also include pathways of uncertain functional relevance. By focusing on well-characterized cerebellar inputs and outputs, the present analysis aims to balance data-driven network analysis with biological interpretability.

In a subsequent step, the extracted subnetwork was subjected to a comprehensive structural characterization. This involved quantifying the connectional architecture using graph-theoretical and topological metrics, aimed at elucidating the organizational principles of cerebellar connectivity. The analysis provided insights into the connectivity density, directionality, modular organization, and potential hub regions within the cerebellar connectome.

**Fig. 1:**
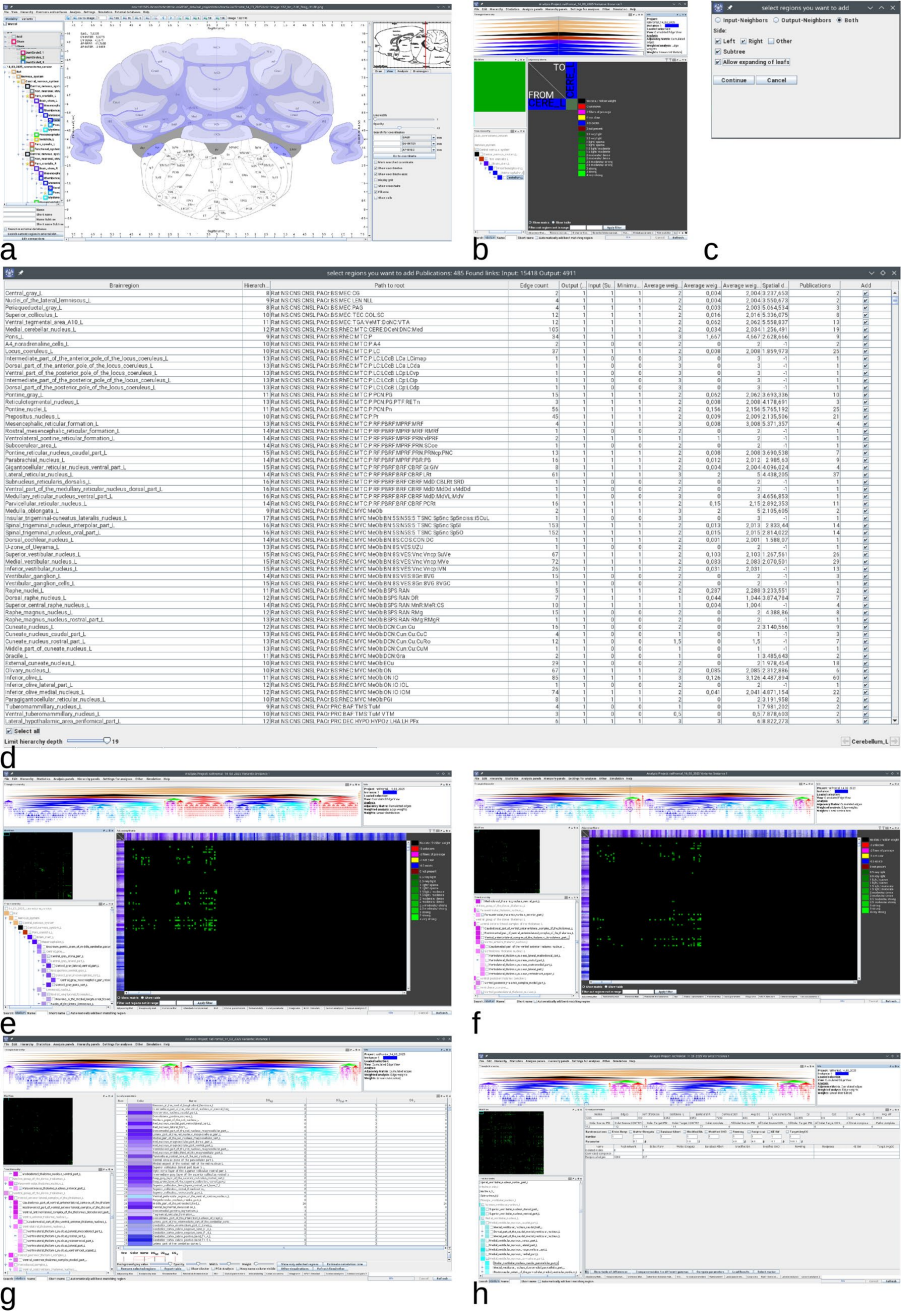
**The 7 steps for configuring a partial connectome from a complete connectome.** **a**) Main window of neuroVIISAS with the current laboratory rat connectome data. A z-plane through the brain stem with the cerebellum in the view of Paxinos’ stereotaxic rat atlas can be seen in the middle window. The bihemispheric ontology can be navigated in the left window. **b**) After opening the “Advanced Connectivity Analysis” window, the triangular representation of the ontology appears with a search region found: “Cerebellum.” **c**) All regions of the cerebellum are searched for by right-clicking and selecting the appropriate settings. **d**) The list of regions found and connected to the cerebellum is displayed in tabular form. **e**) The list of regions found is applied to the ontology, which is expanded accordingly. **f**) The fine-grained division of the spinal regions was reduced because subregions and individual nuclear columns are not of interest for the cerebellar connectome. **g**) Removal of regions without input or output connections from the tables of local connectome parameters. **h**) Final test for a connected component in the global connectome analysis

To generate the cerebellar connectome configuration within the *neuroVIISAS* platform, the following methodological steps were executed:

1. **Initiation of advanced analysis:** The procedure commenced by accessing the *Advanced Analysis* interface via the main menu path (Fig. 1a): $\texttt {Analysis} \rightarrow \texttt {Advanced Analysis}$
2. **Target region identification:** Within the ontology hierarchy of the rat brain connectome, the *cerebellum* was identified and selected by navigating from the root node of the tree of regions (Fig. 1b).
3. **Hierarchical expansion and neighborhood inclusion:** Utilizing the *triangular hierarchical representation*, a depth search was initiated using the function (Fig. 1c-f): $\texttt {Add neighbors of this node}$ The following parameters were specified to ensure comprehensive coverage of regional connectivity:
  **Connection directionality:** Both *Input neighbors* and *Output neighbors* were included.
  **Laterality:** The search was conducted bilaterally by selecting both *Left* and *Right* hemispheric regions.
  **Search scope:** The *Subtree* option was enabled to incorporate all subordinate regions.
  **Leaf expansion:** The setting *Allow expanding of leaves* was activated to ensure terminal nodes (leaf regions) were also fully included in the connectivity analysis.

Regions without incoming or outgoing connections were excluded to ensure that the resulting network forms a single connected component. This step facilitates the computation and interpretation of global graph-theoretical measures.

Finally, using the Local parameters function, all regions were sorted by the number of output connections to compact the connectome. Regions that exhibited no output connections were removed from the dataset (Fig. 1g). The same procedure was applied to eliminate regions without input connections. As a result, each remaining region possesses at least one input and one output connection, ensuring that the entire connectome forms a single, graph-theoretically connected component (Fig. 1h).

This configuration enabled the extraction and visualization of the cerebellar connectome architecture in a detailed and bidirectional manner, supporting both intra- and inter-regional connectivity exploration within the rat connectome.

## Results

### Local Cerebellar Circuits

**Fig. 2:**
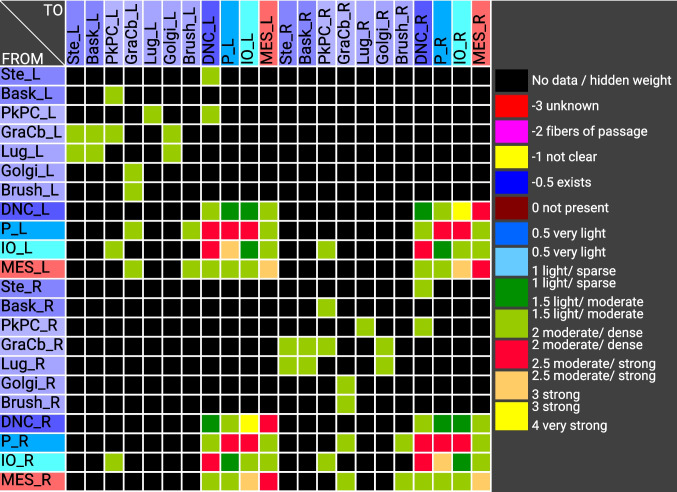
**The adjacency matrix for the bilateral cerebellar microcircuits**. The color scale shows the estimated connection density (connection weights). **Abbreviations**: Ste – stellate neurons; Bask – basket neurons; PkPC – Purkinje cells; GraCb – granule cells; Lug – Lugaro neurons; Golgi – Golgi cells; Brush – unipolar brush neurons; DNC – deep cerebellar nuclei; P – pons; IO: inferior olivary nucleus; MES – spinal cord; left (_L) and right (_R) hemispheric regions

**Fig. 3:**
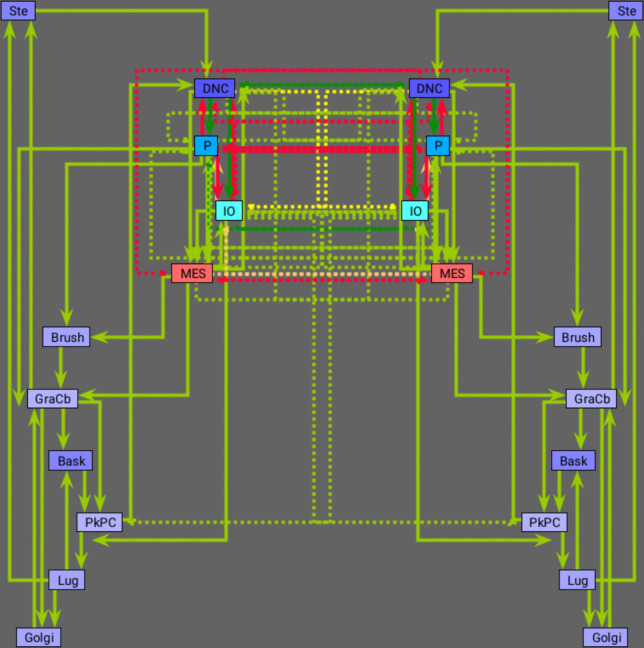
**Representation of the microcircuit adjacency matrix as a bilateral hierarchical network diagram**. The dashed lines indicate contralateral connections–from climbing fibers to deep cerebellar nuclei and Purkinje cells, as well as between extracerebellar nuclei. Mossy fibers remain uncrossed. The color coding of the connections corresponds to the color scale in the previous figure. The color scale shows the estimated connection density (connection weights). Left and right hemispheric regions are shown on the left and right side of the diagram. **Abbreviations**: Ste – stellate neurons; Bask – basket neurons; PkPC – Purkinje cells; GraCb – granule cells; Lug – Lugaro neurons; Golgi – Golgi cells; Brush – unipolar brush neurons; DNC – deep cerebellar nuclei; P – pons; IO: inferior olivary nucleus; MES – spinal cord

The extraction and representation of local cerebellar microcircuit connectivity from the rat connectome is demonstrated in Figs. 2 and 3. The microcircuit adjacency matrix (Fig. 2) encodes directional synaptic relationships among distinct cerebellar regions and neuronal populations. When visualized as a bilateral hierarchical connectome (Fig. 3), this matrix reveals consistent spatial patterns corresponding to known neuroanatomical pathways. The connectivity graph reflects both ipsilateral and contralateral projections, characteristic of cerebellar circuit architecture. Contralateral climbing fiber pathways, originating in the inferior olive and targeting Purkinje neurons and deep cerebellar nuclei, are clearly distinguished as dashed lines. In contrast, mossy fibers–originating from precerebellar sources like the pontine nuclei–display strictly ipsilateral, uncrossed connections. These patterns faithfully recapitulate canonical cerebellar wiring principles, thereby validating the inference of microcircuit connectivity from macroscale data.

The hierarchical connectivity diagram in Fig. 3 captures the canonical architecture of cerebellar microcircuits, enabling the derivation of neuronal cell types, connection pathways, and their functional characteristics. The circuit consists of a core set of neuron classes, each with distinct transmitter phenotypes and specific roles in excitation, inhibition, and modulation of cerebellar signal processing.

The afferent systems are represented by two main fiber types: mossy fibers (MF) and climbing fibers (CF). Mossy fibers originate from precerebellar nuclei and project ipsilaterally into the granular layer, where they form excitatory (glutamatergic) synapses with granule neurons (GraCb) and Golgi neurons (Golgi). Climbing fibers arise from the inferior olive and cross to innervate the contralateral cerebellar hemisphere. They form strong glutamatergic excitatory synapses on Purkinje neurons (PkPC) and send collaterals to the deep cerebellar nuclei (DNC), thereby serving both as signal propagators and modulators of the output timing (Fig. 3).

Granule neurons, the most numerous excitatory neurons, relay mossy fiber input via their parallel fibers (PFs) to multiple downstream targets. PFs synapse on the dendrites of Purkinje neurons, basket neurons (Bask), stellate neurons (Ste), and Golgi neurons. These connections are glutamatergic and excitatory, enabling feedforward propagation of afferent information (Fig. 3).

Purkinje neurons, the sole output neurons of the cerebellar cortex, integrate excitatory input from PFs and CFs, and inhibitory input from interneurons. PkPCs project to the DNC, releasing GABA at their terminals, thus exerting inhibitory control over cerebellar output (Fig. 3).

Inhibitory interneurons modulate cortical processing via precise spatial and temporal gating. Basket neurons and stellate neurons, both excited by PFs, inhibit PkPCs using GABAergic synapses–basket neurons targeting the soma/axon initial segment, and stellate neurons targeting dendrites. Golgi neurons, which also receive excitatory input from PFs and MFs, inhibit granule neurons in glomeruli, providing both feedback and feedforward inhibition.

The connectivity architecture represented in Fig. 3 also supports the inclusion of less prominent but functionally important cerebellar interneurons: Lugaro neurons and unipolar brush neurons (Brush). These neuron types, though sparsely distributed, contribute distinct modulatory and integrative roles within the cerebellar cortex.

Lugaro neurons (Lug) are inhibitory interneurons located beneath the Purkinje neuron layer. They receive inhibitory GABAergic input from Purkinje neuron axon collaterals and possibly from basket neurons, and they extend axonal projections longitudinally across the cerebellar cortex. Their axons target Golgi neurons and basket/stellate neurons, releasing Glycine and GABA, thereby exerting inhibitory effects. The Lugaro neuron pathway provides a feedback loop that modulates the timing and gain of inhibition delivered by other interneurons, effectively fine-tuning granule neuron excitability via indirect Golgi neuron regulation. Though not explicitly delineated in Fig. 3, the underlying hierarchical structure accommodates these lateral interactions, which reflect the cerebellum’s modulatory depth.

Unipolar brush neurons (Brush), found primarily in the vestibulo-cerebellum, are excitatory interneurons residing in the granular layer. They receive glutamatergic input from mossy fibers and amplify sensory input through a prolonged excitatory response. Brushs, in turn, form glutamatergic synapses onto granule neurons and possibly other Brushs, providing excitatory feedforward amplification within local mossy fiber domains. Their inclusion in the connectivity schema is supported by their distinct role in enhancing signal persistence, particularly for vestibular and proprioceptive information. This excitatory relay function is consistent with the connectivity pattern in Fig. 3, where mossy fiber input is locally expanded within the granular layer.

Both Lugaro neurons and Brush neurons contribute specialized computational elements to cerebellar microcircuitry: Lugaro cells via inhibitory control of inhibitory connectomes, and Brushs through excitatory signal amplification. Their roles affirm the complexity and specificity of cerebellar processing, as captured in the connectome diagram. These findings justify their integration into future high-resolution or mesoscale models, particularly for regions involved in balance, eye movement, and autonomic regulation.

The deep cerebellar nuclei, composed predominantly of glutamatergic projection neurons, integrate inhibitory input from PkPCs and excitatory collaterals from MFs and CFs. This integration enables the cerebellum to shape and transmit precisely timed motor and cognitive commands to extracerebellar targets such as the red nucleus and thalamus. The balance between excitation and inhibition at the DNC is a fundamental computational motif in cerebellar function.

Figure 3 further illustrates contralateral connections (dashed lines), particularly the CFs from the inferior olive and reciprocal projections involving the DNC and extracerebellar targets, consistent with bilateral integration. In contrast, MFs exhibit strictly ipsilateral connectivity, reflecting anatomical constraints of sensorimotor afferents.

Together, the connectivity diagram validates the reconstruction of cerebellar microcircuit motifs from the rat connectome. The inclusion of neuron-specific connectivity, synaptic directionality, and transmitter-based polarity underscores the ability to extract functional circuit logic from connectome representations. This lays the groundwork for developing a mesoscale cerebellar connectome that would incorporate mesencephalic, diencephalic, and cortical regions, further elucidating cerebellar contributions to distributed brain processing beyond motor control.

Furthermore, the hierarchical organization observed in the graph supports the notion that intrinsic cerebellar modules are structurally conserved and embedded in a larger bilateral system. The integration of extracerebellar nuclear areas–some of which show contralateral linkage–extends the scope of local circuits and suggests an anatomically grounded logic for interhemispheric processing. The color-coded connectivity strengths, aligned with the scheme from the preceding figure, reinforce the granularity and directionality of the reconstructed connectomes.

These findings confirm that detailed local circuit motifs, including the well-documented divergence of mossy and climbing fiber pathways, can be computationally extracted and reliably represented from a high-resolution connectomic framework. This justifies the use of connectome-based modeling for investigating cerebellar microstructure and provides a platform for cross-validating anatomical data with graph-theoretical representations.

To advance this framework further, we propose the development of a mesoscale cerebellar connectome that incorporates mesencephalic, diencephalic, and cortical structures. Such an expansion would bridge local cerebellar circuitry with broader cerebrocerebellar systems, including the thalamus, basal ganglia, midbrain, and neocortex. This approach could enable a comprehensive understanding of cerebellar integration in sensorimotor control, cognitive modulation, and affective processing. By capturing cross-level interactions from microcircuit to systems-level organization, the mesoscale model would offer a more complete depiction of the cerebellum’s role in whole-brain dynamics.

### Mesoscale Cerebellar Connectome

**Fig. 4:**
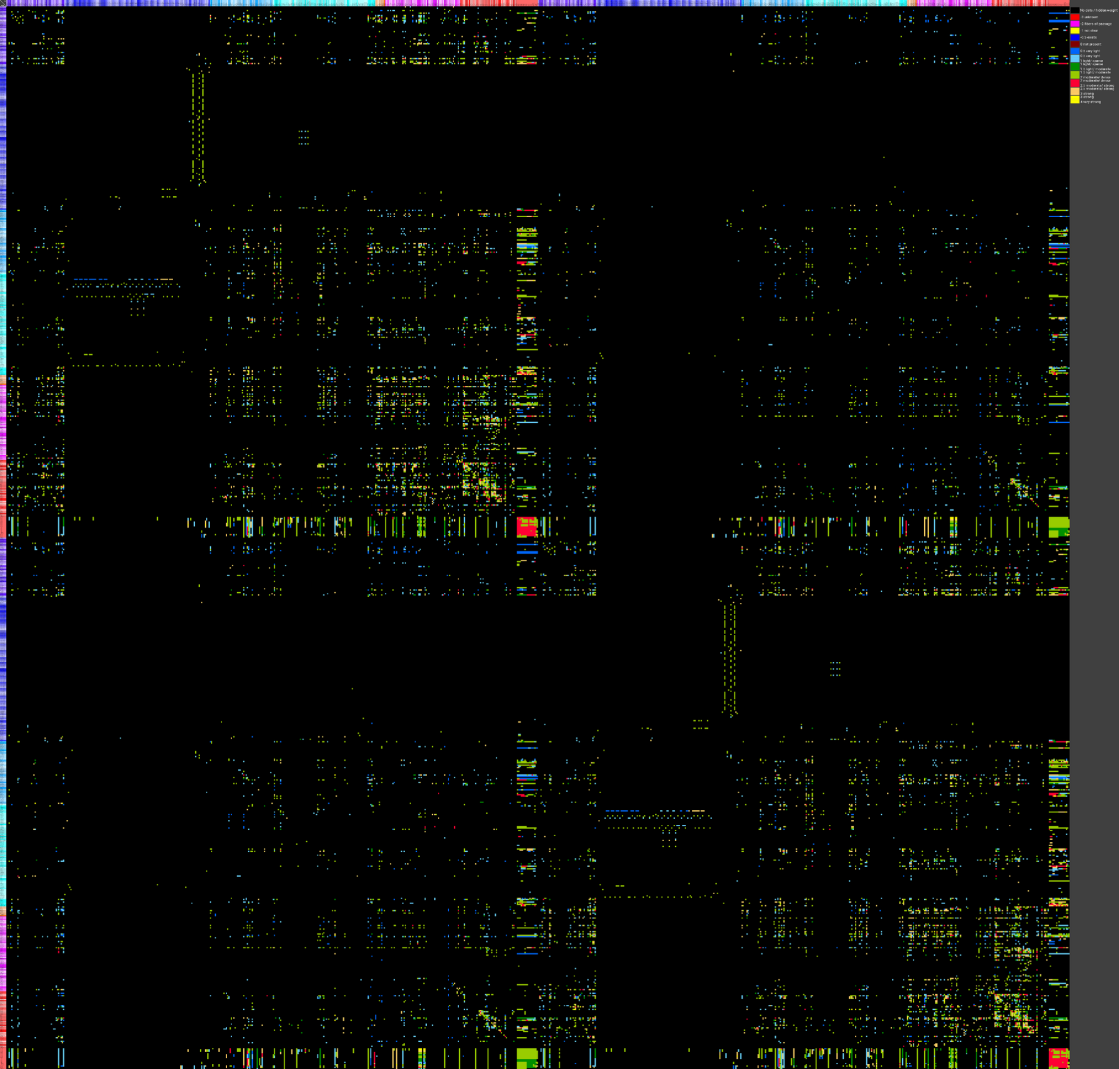
**Weighted adjacency matrix of the bilateral cerebellar connectome**. The matrix encodes pairwise anatomical connectivity between regions of the bilateral cerebellum and associated structures. Rows and columns correspond to distinct regions, with ipsilateral and contralateral hemispheres aligned symmetrically. Each matrix entry reflects the connection strength (weight) and directionality from the region indexed by the row to the region indexed by the column. Color intensity indicates the magnitude of the weighted connection (log-scaled), with brighter tones denoting stronger connectivity. Vertical and horizontal color bars mark anatomical groupings of regions. The top-left and bottom-right quadrants show intrahemispheric connectivity (left-left and right-right), while the off-diagonal blocks (top-right and bottom-left) depict interhemispheric connections. The sparse and asymmetric pattern highlights the structured, non-random nature of cerebellar connectivity and its hemispheric specialization

Global connectome analysis The global connectome analysis of the bilateral cerebellar connectome, derived from the dataset ratFrontal2025, provides comprehensive insights into the topological and functional organization of the cerebellar system. Based on a cumulated edge view using a weighted, directed adjacency matrix with linear weight distribution (Fig. 4). The global connectome analysis of the empirical cerebellar connectome revealed a directed graph comprising 862 nodes and 21,116 edges, including 176 self-referential connections (loops). The average node degree was 48, and the connectome exhibited a line density of 2%, indicating a sparse connectivity pattern. The observed sparsity should be interpreted with caution, as the underlying dataset is derived from aggregated tract-tracing studies and does not represent a fully sampled connectome. Not all possible connections have been experimentally tested or reported, which may contribute to an underestimation of connectivity density. At the same time, sparse connectivity is a well-established characteristic of large-scale brain networks, suggesting that the observed pattern likely reflects both biological organization and sampling limitations. Thus, the reported sparsity reflects a combination of biological network organization and incomplete experimental coverage.

With respect to hemispheric organization, 7,131 connections were ipsilateral within the left hemisphere, 7,127 within the right hemisphere, and 6,858 were contralateral. Additionally, the connectome contained 20,431 directed cycles, highlighting the presence of recurrent and feedback structures.

The empirical cerebellar connectome shows a unique combination of features across multiple topological domains. It is fully connected (no isolated nodes, one connected component). The number of *reciprocal edges* is 3,988, and the reciprocity index is 1.432, reflecting a dense and strongly bidirectional architecture. The connectome exhibits high *degree heterogeneity* (4.182) and maximal *degree distribution entropy* (11.0), pointing to a broad and diversified distribution of connections–indicative of hierarchical structure and hub dominance. The *diameter* of 3.526 and an *average path length* of 2.754 imply compact integration, while the *short self-referential path length* (0.237) and high *directed centrality* (0.958) support the presence of strong feedback pathways (Table 1).

The *central point dominance* (see Appendix  A.0.1) metric reaches extreme magnitudes ($\sim 9.45 \times 10^{27}$), indicating one or more nodes that dominate connectome control and access. Despite this, the *clustering coefficient* (0.315) and *subgraph centrality* (0.272) remain moderately high, suggesting local cohesion around densely connected regions.

Efficiency metrics such as *global efficiency* (0.337), *average closeness* (2.969), and *flow coefficient* (6.675) confirm that the connectome supports both rapid communication and parallel information flow. Structural resilience is evident from its moderate *vulnerability* (0.417) and *transitivity* (0.337), while *modularity* (0.275) and *directed modularity* (0.313) point to the presence of functionally clustered communities. Strikingly, the central point dominance metric reveals vastly higher values than any model ($\sim 10^{27}$), suggesting the presence of extremely influential hub nodes (Table 1). Finally, the connectome displays a high *cyclic coefficient* (11.858).

To assess whether the empirical cerebellar connectome and various surrogate connectomes exhibit scale-free characteristics, we analyzed their degree distributions using a power-law approximation (see Appendix  B). The empirical cerebellar connectome is consistent with *scale-free properties* (e.g., *Δ =0.32*, *γ =0.645*), aligning with known properties of biological neural systems that combine modularity, hierarchical organization, and dynamic flexibility.

Similarity of Network Models to the Empirical Connectome To systematically evaluate how well different generative models capture the structure of the cerebellar connectome, we generated 1000 surrogate networks for each algorithmic model. For each set of surrogates, we computed the mean values of a comprehensive set of global network metrics, enabling robust statistical comparisons across models. Comparing these synthetic networks to the empirical cerebellar connectome serves a dual purpose. First, it allows us to quantitatively assess the capacity of canonical and modified network models to replicate specific topological features observed in biological systems. Second, this comparison acts as a form of null modeling, where deviations between the real and model-generated networks highlight which properties are uniquely constrained by biological organization rather than emerging from general wiring rules. This approach helps isolate structural signatures of the empirical connectome, thereby informing both our understanding of neural architecture and the design of future computational models.

The comparative analysis reveals that no single synthetic network model fully reproduces all features of the empirical cerebellar connectome. However, specific models approximate distinct structural properties more closely than others.

The empirical cerebellar connectome exhibits distinct structural and functional features that set it apart from several canonical synthetic network models. This section provides a detailed comparative narrative of various network properties measured across the empirical connectome and seven model classes: Erdős–Rényi [77], Watts–Strogatz [77], Barabási–Albert [77], Modified BA [15, 61–63, 74, 75], Modified OHO [15, 61–63, 74, 75, 78], Rewiring [79], Reciprocal [80], and KE Small-World (KE SW) [81].

The real cerebellar connectome contains no isolated nodes, a trait shared with most synthetic networks. However, Barabási–Albert and Modified BA models deviate significantly, generating 42 and 18 isolated nodes respectively. All networks maintain a single connected component, preserving global connectivity.

Reciprocity is a defining feature of the cerebellar connectome, with 3988 reciprocal edges and a reciprocity value of 1.432. This structure is exactly preserved in the rewiring and reciprocal models by design. KE SW, while not constructed specifically to preserve reciprocity, exceeds the empirical value with a reciprocity of 1.884. Conversely, Erdõs–Rényi and Watts–Strogatz networks fail to capture this feature, reflecting poor modeling of directionality and feedback.

The empirical network exhibits high heterogeneity (4.182), indicating a wide variation in node connectivity. Modified BA surpasses this with a heterogeneity of 4.747, suggesting an even more skewed degree distribution. The degree distribution entropy is highest in the empirical network (11.0), a reflection of its complex and diverse connectivity profile. All models score substantially lower, with KE SW showing the least entropy (3.735), indicating oversimplified degree patterns.

The empirical network has a moderate diameter of 3.526 and an average path length of 2.754. Most synthetic models display shorter paths, implying overly efficient or simplified wiring. KE SW, in particular, has the shortest diameter (2.166), which may overestimate the connectome’s communication efficiency. Self path length is also noteworthy–0.237 in the emoirical network versus 0.722 in KE SW–suggesting excessive self-looping in the latter.

The average centrality in the empirical connectome is 0.326, reflecting balanced hubness. KE SW, however, inflates this to 1.381, suggesting unrealistic dominance by a few nodes. The CPD is highest in the empirical connectome ($9.45 \times 10^{27}$), suggesting a strong hub structure where communication paths are heavily concentrated through a few key nodes. Among surrogate models, KE SW shows the highest CPD ($4.64 \times 10^{22}$), consistent with its scale-free and hierarchical organization. In contrast, Erdõs–Rényi networks exhibit minimal CPD ($6.19 \times 10^7$), reflecting their homogeneous and unstructured topology. Subgraph centrality is also notably elevated in KE SW (0.479) and Modified OHO (0.340), likely due to dense local clustering and repeated motifs surrounding dominant nodes.

The empirical connectome has a clustering coefficient of 0.315. Watts–Strogatz (0.556), Modified OHO (0.528), and KE SW (0.545) exceed this value, potentially overemphasizing local clustering. The average flow coefficient in the real connectome is 6.675; KE SW inflates this drastically to 19.133. Small-worldness is best captured by Modified OHO (0.538) compared to the empirical value of 0.294, whereas Erdõs–Rényi and Barabási–Albert perform poorly in this respect.

The empirical modularity is 0.275, with Modified OHO achieving the highest score (0.617), reflecting a strong community structure. Watts–Strogatz also performs well (0.568). Directed modularity follows a similar pattern: only Watts–Strogatz and Rewiring approach the empirical value (0.313), while other models remain low.

Global efficiency in the empirical connectome is 0.337. KE SW surpasses this (0.482), which may overstate the connectome’s actual communicability. The average closeness is highest in the empirical connectome (2.969), supporting its high integration. Vulnerability is also substantial in the empirical connectome (0.417), indicating sensitivity to targeted disruptions. Modified OHO (0.661) and KE SW (0.713) reflect similar trends but may exaggerate this fragility.

The cyclic coefficient in the real connectome is 11.858. Modified OHO exceeds this slightly (13.246), indicating that it best preserves motifs such as feedback loops. Other models fall short in capturing the richness of cyclic connectivity.

The higher-order measures such as search information, knotty-centeredness, and scale-free indicators (*Δ *, *γ *) (see Appendix  B) were uniformly valued at 0.122 across all models and the empirical connectome. However, considering the scale-free property parameters–specifically *Δ *, *γ *, and *α *–we gain a more nuanced view of power-law adherence. The empirical cerebellar connectome shows moderate scale-free characteristics with *Δ = 0.32*, *γ = 0.645*, and *α = 0.061*, indicating a partial fit to power-law behavior with mild decay and non-negligible exponent. Modified BA and Barabási–Albert models demonstrate stronger scale-free tendencies (*Δ = 0.31* to 0.58), with *γ * values around 0.8–1.26 and *α * values ranging from 0.117 to 0.937. In contrast, Modified OHO shows exaggerated scaling with *α = 48.246*, likely an effect of structural constraints. KE SW presents the most extreme parameters (*γ = 0.952*, *α = 0.223*), reinforcing earlier observations that it overemphasizes hierarchical bottlenecks. Erdõs–Rényi and Watts–Strogatz models remain inconsistent with scale-free structure, showing either low or negative *γ * (e.g., WS: *γ = -0.979*), and *α = 0* or near zero, indicating a poor fit to power-law distributions. When considering exponential approximations, the empirical connectome maintains a moderate fit with *Δ = 0.43*, *γ = 167.508*, and *α = 0.008*, suggesting a shallow exponential decay. KE SW again stands out with a steep exponential profile (*Δ = 0.82*, *γ = 379.598*, *α = 0.004*), highlighting over-regularization. Erdõs–Rényi and Watts–Strogatz models display unstable or extreme *γ * values (e.g., WS: *γ = -219.427*), confirming their unsuitability for modeling higher-order topologies. In summary, the enhanced scale-free and exponential parameters provide clearer differentiation between models. The empirical connectome lies somewhere between a scale-free and exponential regime, while KE SW and Modified BA exaggerate scale-freeness, and Erdõs–Rényi and Watts–Strogatz fail to capture it meaningfully.

In conclusion, no single surrogate model fully reproduces the complete profile of the empirical cerebellar connectome. Nevertheless, Modified OHO emerges as the closest match overall, capturing key properties such as directedness, clustering, path length, small-worldness, modularity, and motif richness. The Rewiring and Reciprocal models also perform well in preserving reciprocity and degree structure. Modified BA and KE SW replicate scale-free characteristics but tend to overfit, leading to unrealistic centralization or excessive clustering. These results underscore the complexity of the biological connectome and the challenges of capturing its features with any single network model.

**Table 1:** **Global network parameters of the cerebellar connectome and surrogate models**. Comparison of mean connectome metrics across various network models (1000 repetitions) and the empirical cerebellar connectome. Erdõs–Rényi: Random network where each possible edge exists with fixed probability; produces graphs with a Poisson degree distribution [82]. Watts–Strogatz: Small-world network generated by random rewiring of a regular lattice; combines high clustering with short average path lengths [83]. Barabási–Albert: Scale-free network model with growth and preferential attachment; generates power-law degree distributions [84]. OHO: Ozik-Hunt-Ott small-world rewiring algorithm adapted for *directed* networks with a fixed number of edges [78]. Rewiring: Random rewiring of the empirical network while preserving the in-degree and out-degree of each node; used as a degree-preserving null model. Reciprocal: Network rewired to preserve the number of *reciprocal edges* observed in the empirical connectome. KE SW: Growing scale-free network model with small-world characteristics, preserving clustering while maintaining scale-free topology [81]

Metric	Real	Erdos Renyi	Watts-Strogatz	Barabasi-Albert	Modified BA	Modified OHO	Rewiring	Reciprocal	KE SW
Isolated nodes	0.000	0.000	0.000	42.000	18.105	0.000	0.000	0.000	0.000
Connected components	1.000	1.000	1.000	1.000	1.000	1.000	1.000	1.000	1.000
Reciprocal edges	3988.000	302.552	6877.455	1503.042	1729.474	6616.209	2260.276	3988.000	7399.632
Reciprocity	1.432	0.141	0.068	0.907	0.948	0.522	1.432	1.432	1.884
Heterogeneity	4.182	3.322	2.597	3.965	4.747	4.141	4.182	4.182	3.413
Degree distribution entropy	11.000	4.000	4.000	4.042	5.316	5.433	6.586	6.733	3.735
Diameter	3.526	2.456	2.716	2.345	2.489	3.089	2.696	2.739	2.166
Average path length	2.754	2.189	1.796	1.974	2.172	1.796	2.549	2.319	1.796
Avg. path length (self)	0.237	0.026	0.012	0.263	0.233	0.078	0.258	0.230	0.722
Centrality	0.326	0.027	0.014	0.343	0.306	0.130	0.326	0.326	1.381
Centrality Directed	0.958	0.522	0.519	0.962	0.945	0.958	0.940	0.939	0.995
Central point dominance	9.45e27	6.19e7	7.62e7	1.50e16	2.74e17	2.31e13	2.70e20	1.33e18	4.64e22
Average subgraph centrality	0.272	0.028	0.306	0.087	0.096	0.340	0.288	0.257	0.479
Average cluster coefficient	0.315	0.257	0.556	0.263	0.266	0.528	0.274	0.306	0.545
Average flow coefficient	6.675	1.000	9.746	3.213	3.324	9.512	9.242	8.120	19.133
Small-worldness	0.294	0.117	0.495	0.111	0.106	0.538	0.086	0.093	0.424
Modularity	0.275	0.107	0.568	0.099	0.096	0.617	0.075	0.081	0.446
Directed Modularity	0.313	0.028	0.379	0.089	0.099	0.274	0.224	0.237	0.107
Transitivity	0.337	0.434	0.391	0.410	0.398	0.356	0.403	0.398	0.482
Global Efficiency	0.337	0.434	0.392	0.410	0.398	0.356	0.403	0.398	0.482
Average Closeness	2.969	2.307	2.555	2.440	2.512	2.811	2.483	2.513	2.078
Harmonic mean	0.024	0.001	0.000	0.005	0.004	0.006	0.009	0.009	0.026
Vulnerability	0.417	0.090	0.443	0.312	0.309	0.661	0.487	0.442	0.713
Local Efficiency	-0.016	-0.002	0.085	-0.020	-0.022	0.359	-0.156	-0.126	-0.127
Directed assortativity	0.120	0.114	0.134	0.118	0.118	0.139	0.134	0.131	0.148
Cyclic coefficient	11.858	9.286	10.770	8.872	9.428	13.246	11.382	11.529	11.122
Average search information	0.122	0.003	0.004	0.313	0.101	0.306	0.197	0.198	0.566
Knotty-centredness	0.122	0.003	0.004	0.313	0.101	0.306	0.197	0.198	0.566
Scale-Free property	*Δ =0.32* *γ =0.645* *α =0.061*	*Δ =1.89* *γ =0.151* *α =0.026*	*Δ =4.02* *γ =-0.979* *α =0*	*Δ =0.58* *γ =1.258* *α =0.937*	*Δ =0.31* *γ =0.822* *α =0.117*	*Δ =0.21* *γ =2.147* *α =48.246*	*Δ =0.32* *γ =0.645* *α =0.061*	*Δ =0.32* *γ =0.645* *α =0.061*	*Δ =0.73* *γ =0.952* *α =0.223*
Exponential approx.	*Δ =0.43* *γ =167.508* *α =0.008*	*Δ =1.89* *γ =66.296* *α =0.031*	*Δ =4.01* *γ =-219.427* *α =0.015*	*Δ =0.55* *γ =97.901* *α =0.010*	*Δ =0.19* *γ =75.005* *α =0.013*	*Δ =0.30* *γ =35.427* *α =0.048*	*Δ =0.43* *γ =167.508* *α =0.008*	*Δ =0.43* *γ =167.508* *α =0.008*	*Δ =0.82* *γ =379.598* *α =0.004*

#### Local Connectome Analysis

**Fig. 5:**
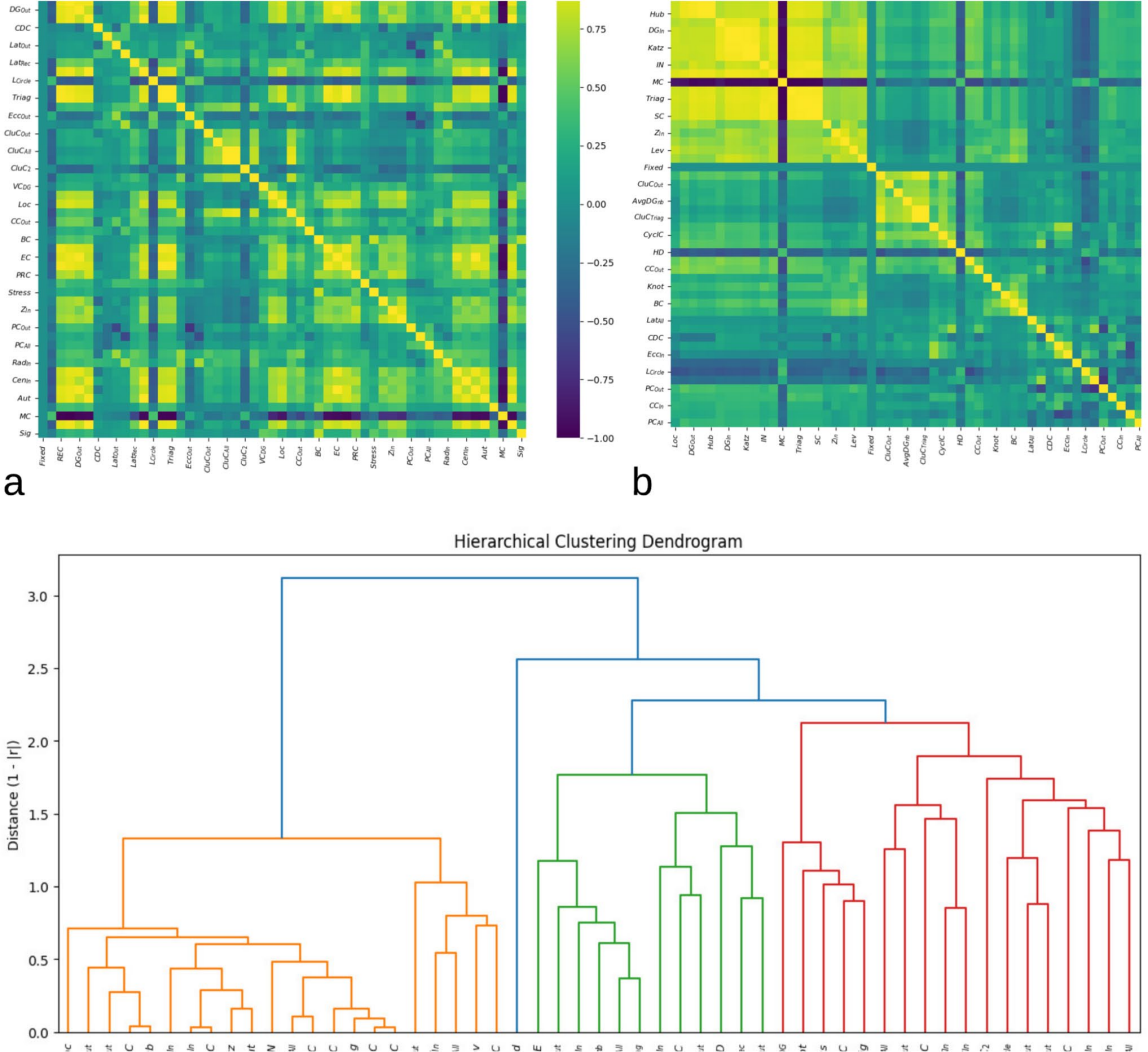
**Correlation and clustering of local network metrics.** **a**) Correlation matrix showing strong dependencies among metrics. **b**) Hierarchical clustering based on correlation distance (*1 - |r|*). **c**) Reordered correlation matrix highlighting clusters of redundant metrics

To reduce redundancy among the large number of computed local network metrics (*n=51*), we performed a systematic correlation and clustering analysis (Fig. 5). Pairwise correlations revealed extensive dependencies between many commonly used network measures, indicating that multiple metrics capture overlapping structural properties of the connectome. Many commonly used network metrics, including multiple centrality and controllability measures, were found to be strongly correlated and therefore do not provide independent information.

Hierarchical clustering based on correlation distance (*1 - |r|*) identified distinct groups of highly correlated metrics. These clusters correspond to major structural dimensions of local network organization, including connectivity strength, network integration, local topology, modular participation, and network vulnerability. This analysis allowed us to reduce the full metric set to a small number of representative descriptors, minimizing redundancy while preserving the key structural information (see Appendix A.0.1 for full analysis).

Based on this reduction, we focused on a subset of core metrics capturing complementary aspects of cerebellar network organization. Connectivity strength was characterized by degree, identifying highly connected regions such as the *pedunculopontine tegmental nucleus*, *pontine reticular nuclei*, and *parabrachial nuclei*. Network integration was assessed using Katz centrality, reflecting the involvement of nodes in multi-step communication pathways across the connectome.

Local circuit organization was captured by clustering coefficients and triangle counts, indicating the presence of densely interconnected microcircuits. Cerebellar cortical regions exhibited higher clustering and lower participation, consistent with locally specialized processing. In contrast, brainstem and deep cerebellar nuclei showed lower clustering and higher participation, indicating a role in cross-modular integration.

The participation coefficient further revealed a gradient from modular specialization to integrative connectivity. Regions such as the parabrachial and pontine nuclei displayed intermediate participation values, suggesting a role in coordinating information flow between functional subsystems.

Network vulnerability was quantified using the Sig parameter, which measures the impact of node removal on global network efficiency. Regions with high Sig values, such as the *infralimbic cortex* and *lumbar segments*, indicate nodes whose removal substantially alters connectome organization, highlighting their importance for maintaining global connectivity.

Together, these results reveal a structured organization of the cerebellar connectome, characterized by a transition from locally specialized regions to highly connected integrative hubs. This organization supports the role of the cerebellum as both a modular processing system and a central integrator of distributed brain networks.

Local Connectomics of Cerebellar Regions The reduced set of local network metrics reveals consistent and biologically meaningful patterns across cerebellar and brainstem regions (Table 5). A prominent feature is the strong bilateral symmetry, with homologous regions such as the *pedunculopontine tegmental nucleus*, *pontine reticular nuclei*, and *parabrachial nuclei* exhibiting nearly identical values across all selected metrics. This symmetry reflects the fundamental bilateral organization of cerebellar processing.

Highly connected and integrative regions are characterized by simultaneously elevated degree and Katz centrality. For example, the *pedunculopontine tegmental nucleus* shows the highest values (DG$_{\text {All}}$ = 287, Katz *≈ * 293), indicating both strong direct connectivity and a central role in multi-step communication pathways. Similarly, the *pontine reticular nuclei* and *parabrachial nuclei* combine high degree (DG$_{\text {All}}$ = 224–234) with substantial Katz centrality (*≈ * 241–246), supporting their role as integrative relay structures.

Local circuit organization differs markedly between regions. Nodes with high triangle counts (e.g., lumbar segments: Triag *≈ * 26983) indicate dense local motif structure, whereas lower clustering coefficients in highly connected brainstem nuclei suggest a shift from local processing toward distributed integration. In contrast, cortical regions exhibit comparatively higher clustering relative to their connectivity, consistent with locally specialized processing.

The participation coefficient reveals a gradient from modular specialization to integrative connectivity. Brainstem relay nuclei such as the *pontine* and *parabrachial nuclei* show intermediate participation values (PC$_{\text {All}} \approx 0.50$–0.55), indicating balanced intra- and inter-modular connectivity. Lower participation values in spinal and segmental regions (PC$_{\text {All}} \approx 0.39$–0.47) suggest more confined, module-specific roles.

Network vulnerability, captured by the Sig parameter, identifies regions with a strong influence on global connectome organization. High Sig values in regions such as the *infralimbic cortex* (Sig *≈ 0.52*) and *lumbar segments* (Sig *≈ 0.39*) indicate that their removal substantially alters global efficiency. Notably, vulnerability is not strictly coupled to degree, suggesting that highly connected nodes are not necessarily those with the greatest impact on global network stability.

Together, these findings reveal a hierarchical organization of the cerebellar connectome, ranging from locally specialized modules to highly connected integrative hubs. Brainstem and deep cerebellar nuclei act as central relay structures combining high connectivity, multi-step integration, and moderate modular participation, while cortical and segmental regions exhibit more localized and functionally specialized network roles. A more detailed description of the full set of local network metrics and their interdependencies is provided in Appendix A.0.1.

**Table 2:** **Reduced set of local network parameters of high-ranked cerebellar connectome regions**. Top regions sorted by average rank across all metrics. The table presents a non-redundant subset of representative local network parameters derived from correlation and clustering analysis. $DG_{\text {All}}$: Degree centrality (total number of connections), Katz: Katz centrality reflecting multi-step connectivity, Triag: Number of triangles (local motif count), CluC$_2$: Hierarchical clustering coefficient (2nd order), $PC_{\text {All}}$: Participation coefficient, Sig: Impact of node removal on global efficiency

Region	DG$_{\text {All}}$	Katz	Triag	CluC$_2$	PC$_{\text {All}}$	Sig
Pedunculopontine_tegmental_nucleus_R	287	293.25	22341	0.0103	0.5467	0.1877
Pontine_reticular_nucleus_oral_part_L	224	246.49	17149	0.0136	0.5488	0.1274
Pontine_reticular_nucleus_oral_part_R	224	246.49	17150	0.0136	0.5488	0.1274
Pedunculopontine_tegmental_nucleus_L	287	293.25	22339	0.0104	0.5467	0.1876
Nucleus_of_Darkschewitsch_R	127	170.91	7954	0.0225	0.5033	0.2155
Nucleus_of_Darkschewitsch_L	127	170.91	7953	0.0225	0.5033	0.2152
Parabrachial_nucleus_medial_L	234	241.54	16499	0.0179	0.5085	0.1640
Parabrachial_nucleus_medial_R	234	241.55	16499	0.0179	0.5085	0.1640
Lateral_paragigantocellular_nucleus_L	209	235.03	16719	0.0160	0.4816	0.1277
Lateral_paragigantocellular_nucleus_R	209	235.03	16719	0.0160	0.4816	0.1277
Laterodorsal_tegmental_nucleus_R	277	264.81	20848	0.0139	0.4813	0.1703
Laterodorsal_tegmental_nucleus_L	277	264.81	20846	0.0139	0.4813	0.1703
Bed_nucleus_of_the_stria_terminalis_R	276	242.18	15865	0.0235	0.4633	0.3405
Bed_nucleus_of_the_stria_terminalis_L	276	242.18	15864	0.0235	0.4633	0.3405
Posterior_hypothalamic_area_R	191	205.20	10756	0.0213	0.5225	0.1123
Posterior_hypothalamic_area_L	191	205.20	10755	0.0213	0.5216	0.1123
Lumbar_segments_R	329	307.65	26983	0.0119	0.4675	0.3924
Infralimbic_cortex_L	267	262.86	17371	0.0116	0.4864	0.5225
Infralimbic_cortex_R	267	262.86	17371	0.0116	0.4864	0.5225
Lumbar_segments_L	329	307.65	26983	0.0119	0.4634	0.3923
Thoracal_segment_3_R	228	266.37	20893	0.0154	0.3984	0.1246
Thoracal_segment_3_L	228	266.37	20893	0.0154	0.3984	0.1246
Cingulate_cortex_L	278	183.74	17233	0.0106	0.4647	0.3589
Cingulate_cortex_R	278	183.72	17235	0.0106	0.4647	0.3593
Cuneiforme_nucleus_L	187	201.37	12395	0.0215	0.5284	0.2013
Cuneiforme_nucleus_R	187	201.37	12395	0.0215	0.5284	0.2013
Thoracal_segment_2_R	222	262.48	20513	0.0152	0.3903	0.1061
Thoracal_segment_2_L	222	262.48	20513	0.0152	0.3903	0.1061
Thoracal_segment_1_L	243	256.04	21542	0.0138	0.4232	0.1274
Cervical_segment_1_R	293	280.75	23956	0.0124	0.4544	0.4254

#### Motif Analysis

**Fig. 6:**
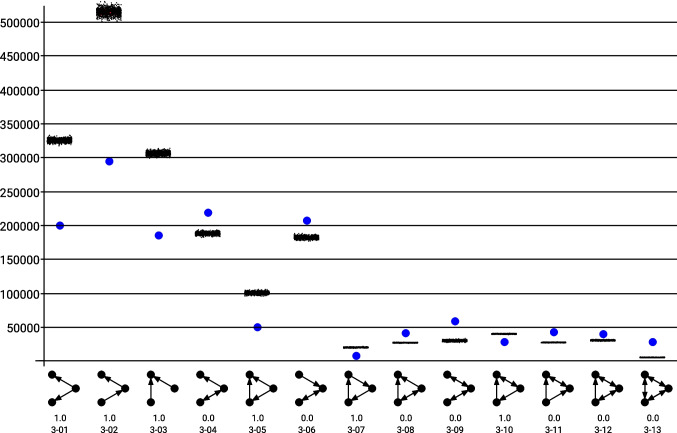
Motif distribution in the empirical cerebellar connectome (blue points) and 1000 rewiring simulations (black points)

The motif (see Appendix  D) distribution in the empirical cerebellar connectome, visualized in Fig. 6, reveals notable deviations from the distribution obtained through 1000 rewired simulations. These differences are quantitatively supported by the statistical data presented in Table 3, which includes various motif-specific empirical counts (EC1, EC2, EC3), rewiring means (Rm), *p*-values, *z*-scores, and standard deviations.

Motifs 3-01 through 3-03 represent feedforward-like structures with high empirical counts (EC1) and similarly high rewired means Table 3. Their *p*-values of 1.00 and strongly negative *z*-values (e.g., *z = -51.61* for motif 3-01) indicate that although these motifs are common in the empirical connectome, they are also prevalent in randomized networks. Consequently, they are not statistically overrepresented and likely reflect baseline connectivity patterns.

In contrast, motifs such as 3-04, 3-06, 3-08, 3-09, 3-11, 3-12, and especially 3-13 exhibit significant deviations. These motifs display either significantly higher or lower empirical counts compared to their respective rewired means, accompanied by values of *p<0.001* and large absolute *z*-scores. For instance, motif 3-13, with a relatively modest empirical count of 27,297, stands out with a very low rewired mean (4,972,225) and a striking *z*-value of 94.64, indicating strong deviation from the degree-preserving random model. Similarly, motif 3-11 has a high positive *z*-value (52.15), pointing to its non-random organization in the connectome.

These statistically significant motifs may reflect structural constraints not captured by the applied null model, including biological or spatial organization in the cerebellar connectome, which are not reproduced by random rewiring. The large positive *z*-values further imply that these motifs occur far more frequently than expected by chance, and their low standard deviations reinforce the robustness of these observations across simulations.

Interestingly, some motifs, such as 3-04 and 3-06, show positive *z*-values and values of *p<0.001* despite moderate empirical counts. This implies that even relatively moderate empirical frequencies can be statistically significant if the motif is rare in the null model. In contrast, motifs like 3-07 and 3-10 demonstrate low empirical counts yet still have *p*-values of 1.00, indicating underrepresentation with respect to the random model and thus lack functional significance in the context of motif enrichment.

Altogether, the diagram and accompanying table collectively highlight specific connectome motifs that deviate meaningfully from random wiring statistics, offering insights into the non-random organization of the cerebellar connectome.

It should be noted that the applied rewiring models preserve degree distribution but do not account for spatial embedding, anatomical constraints, or developmental processes. Therefore, deviations from the null model should be interpreted as deviations from random connectivity under these constraints, rather than direct evidence of functional optimization. Such deviations should therefore be interpreted as indicators of structural organization beyond degree constraints, rather than as direct evidence for specific functional or evolutionary optimization principles.

**Table 3:**
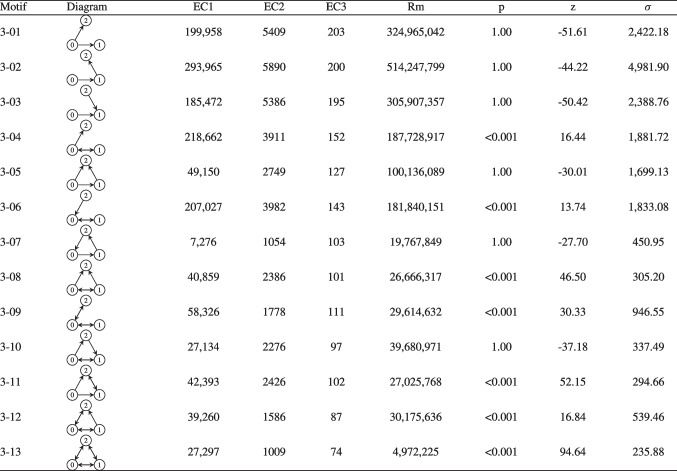
**Motif frequencies in the empirical cerebellar connectome**. Motif frequencies in the empirical cerebellar connectome vs. 1000 rewired simulations. EC1: empirical count with repetitions. EC2: empirical count without multiple use of edges, EC3: empirical count without multiple use of nodes, Rm: Rewiring mean, *σ *: standard deviation

#### Community Analysis

Community detection offers a powerful framework for exploring the modular organization of the cerebellar connectome and provides insights that extend beyond traditional anatomical subdivisions. The cerebellum, though historically associated primarily with motor coordination, is now recognized to play crucial roles in a wide range of cognitive, emotional, and sensorimotor functions. Understanding how its internal connectivity supports this functional diversity requires analytical tools that can reveal hidden structure in complex networks.

One primary motivation for applying community detection is its ability to uncover *functional subdivisions beyond anatomy*. While the cerebellum is anatomically organized into lobules and hemispheres, data-driven community detection allows for the identification of modules based on actual connectivity patterns–both within the cerebellum and between the cerebellum and cerebral cortex. These modules may correspond to functionally distinct subsystems, such as those involved in motor execution, working memory, or affective regulation, which are not necessarily aligned with anatomical boundaries.

Furthermore, community detection provides a formal method to examine the balance of *integration and segregation* within the cerebellar network. Segregation refers to the degree to which cerebellar regions form self-contained clusters that process information independently, whereas integration refers to the presence of connector nodes or hubs that link otherwise distinct modules. This modular structure supports the cerebellum’s role in orchestrating domain-specific computations while simultaneously enabling cross-domain coordination–for example, in tasks that require coupling of motor actions with cognitive planning.

Another advantage of community detection is its capacity to reveal *hierarchical or overlapping organization*. The cerebellum may possess nested modules–for instance, finer subdivisions within a lobule that contribute to broader zones of function. Algorithms such as the Louvain method can identify such hierarchical modularity, offering insights into how cerebellar processing unfolds across multiple scales. This multi-scale organization is critical for understanding how local computations relate to global cerebellar dynamics.

Importantly, community detection aids in *linking structure to function and pathology*. By comparing cerebellar modules derived from structural connectomes with patterns observed in functional imaging (e.g., resting-state fMRI or task-based activations), researchers can better understand the structure–function correspondence of cerebellar circuits. Moreover, modular analysis may help identify specific cerebellar communities that are selectively affected in neurological and psychiatric disorders, such as spinocerebellar ataxia, autism spectrum disorder, or schizophrenia, where distinct patterns of cerebellar dysconnectivity have been reported. The methodological approach used to reliably detect communities in the cerebellar connectome is explained in Appendix G.

**Fig. 7:**
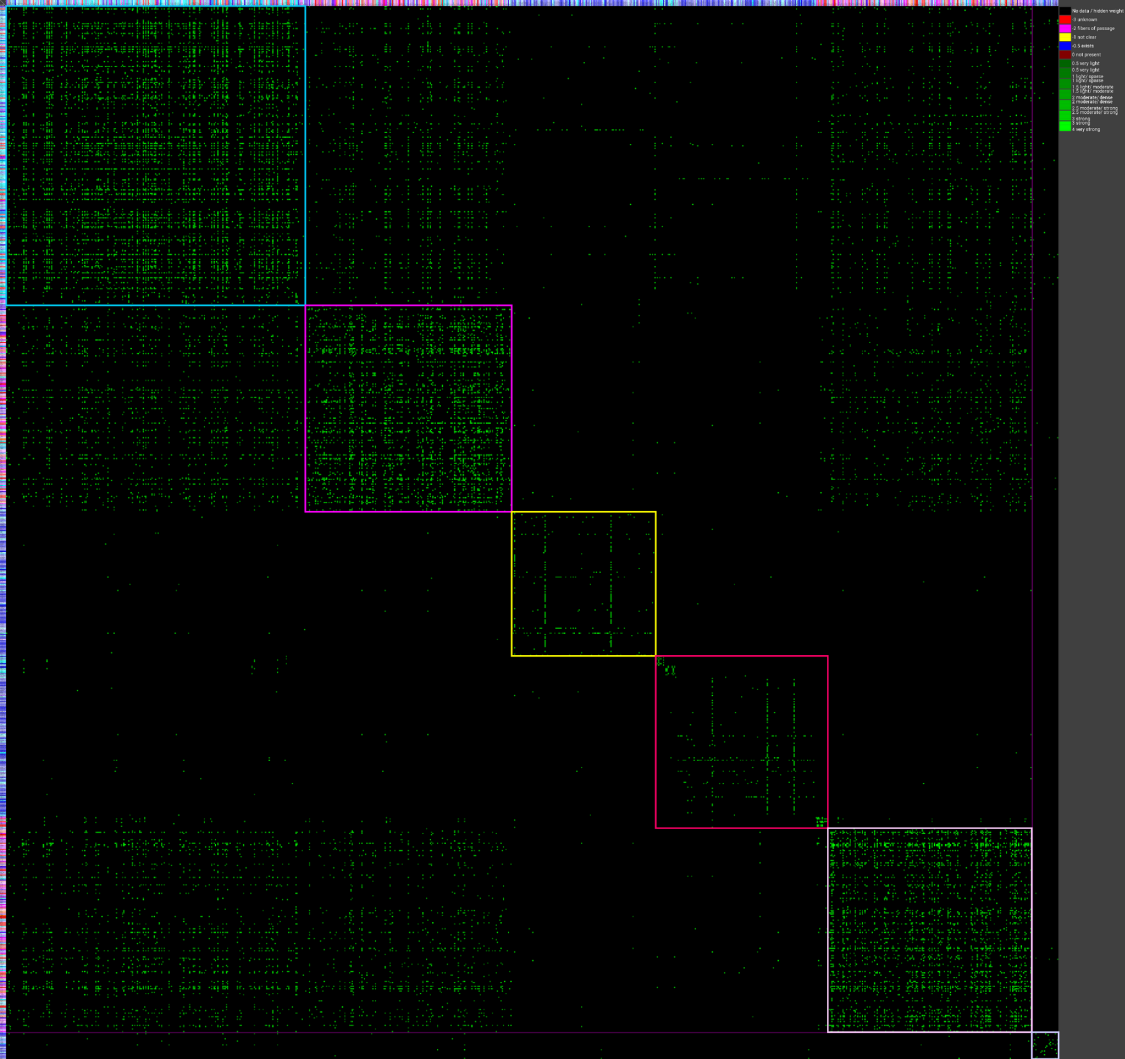
**Louvain modularity analysis of the full brain connectome with consensus clustering.** The matrix visualization shows the reordered adjacency or similarity matrix of the brain network based on the results of a Louvain modularity analysis followed by consensus clustering. Each row and column corresponds to a neuroanatomical region, with rows and columns arranged to reflect modular groupings. Six distinct clusters were identified and are visually separated by colored borders. These clusters, color-coded as light blue, purple, yellow, red, white, and magenta, represent consensus modules that exhibit high intra-cluster connectivity and relatively sparse inter-cluster connectivity, indicating robust community structure. The modular decomposition was derived from repeated Louvain runs with a consensus clustering algorithm to ensure stability of the detected modules. This modular architecture highlights the presence of topologically and potentially functionally distinct subsystems within the brain connectome, including dense modules likely corresponding to sensorimotor, associative, limbic, and cerebellar components

The matrix shown Fig. 7 has been reorganized based on the results of a Louvain modularity analysis, which partitions the network into communities by maximizing the modularity function * Q *. This reordering was applied to a symmetric network matrix–such as a structural connectivity matrix, generalized topology overlap matrix (GTOM), or functional similarity matrix–where rows and columns represent identical neuroanatomical regions.

This reorganization enhances the visibility of modular structure within the network and facilitates the interpretation of both the strength and specificity of intra- and inter-modular relationships. The color-coded outlines demarcate the consensus communities identified across multiple iterations of the Louvain algorithm, stabilizing variability and improving reliability of community assignments. The resulting modular architecture provides evidence for a non-random, functionally meaningful organization of the network, with distinct subsystems likely reflecting specialized processing domains within the brain.

The six consensus clusters identified through Louvain modularity analysis were further characterized by tracing each region’s topographical hierarchy. This analysis preserved the original anatomical nesting of regions in a tree-like format, allowing for a detailed understanding of the structural and functional coherence within each cluster.

The area outlined by a blue rectangle in the community matrix delimited cluster 1 (internally referred to as cluster_2) (Fig. 7). It predominantly comprises regions associated with the left somatosensory and entorhinal cortices. The most frequent top-level anatomical entries include *S1BF* (barrel field of primary somatosensory cortex), *Ent* (entorhinal cortex), and *Cg* (cingulate cortex), with additional contributions from *Pir* (piriform cortex) and *CA1* (hippocampal subfield CA1). This cluster likely represents a sensorimotor-limbic module, integrating tactile input with spatial and associative memory processing (see Appendix A for abbreviations).

Cluster 2, marked in magenta (internally designated as cluster_703), then follows on the main diagonal. The contralateral counterpart to cluster 1, is composed of analogous regions in the right hemisphere: *S1BF*, *Ent*, *Cg*, *Pir*, and *CA1*. The mirrored composition suggests a strong bilateral symmetry in cortical-limbic integration, reinforcing the idea of functionally homologous modules across hemispheres.

Cluster 3, marked with yellow (internally designated as cluster_171), then follows on the main diagonal. Cluster 3 is dominated by lateral and septal segment structures, especially *LSeg*, *LSeg*, *SSeg*, *SSeg*, and *TSegM*, which are typically involved in thalamic relay and multisensory integration. The dense bilateral representation and prominence of segmental relay zones indicate a functional role in coordinating ascending sensory streams and modulating cortical access to subcortical input.

Cluster 4, marked in red (internally designated as cluster_418), then follows on the main diagonal. Cluster 4 includes nuclei from the auditory and trigeminal brainstem pathways, such as *NLL*, *NLL* (nuclei of the lateral lemniscus), and *Sp5CC* (spinal trigeminal complex). The inclusion of *OT* and *OT* (optic tract regions) suggests this cluster may represent a multimodal sensory gateway, particularly focused on integrating auditory and somatosensory information within the brainstem.

Cluster 5, marked in pink (internally designated as cluster_562), then follows on the main diagonal. Cluster 5 appears to be a cerebellar-brainstem module. It contains subnuclei such as *CeZC3*, *CeZA*, and *Pr5ca*, which are associated with proprioceptive feedback and cerebellar input processing. The left-lateralized dominance and presence of trigeminal subfields indicate a role in coordinating fine somatosensory motor integration, possibly with cerebellar output pathways.

The last cluster 6 on the main diagonal, marked in light purple (internally referred to as cluster_680). Cluster 6 is composed of a smaller set of highly specific structures, such as *DLPr*, *DLPr*, *MedV*, and *CopM*, which are typically associated with deep mesencephalic and pontine nuclei. These regions may participate in reticular formation circuits or serve as integrative hubs linking motor coordination and visceral autonomic functions.

In summary, the hierarchical tree-structured analysis reveals that the six Louvain consensus clusters exhibit coherent topographical and functional themes, ranging from bilateral cortical-limbic integration (Clusters 1 and 2), through thalamic-segmental relay zones (Cluster 3), to multimodal sensory processing (Cluster 4), cerebellar-somatosensory coordination (Cluster 5), and brainstem motor-autonomic hubs (Cluster 6). This underscores the ability of data-driven modularity analysis to uncover biologically meaningful subdivisions within the connectome.

#### Connectivity Matching Analysis

Connectivity matching matrices are a valuable tool in connectome analysis, as they quantify the similarity between neurons or regions based on their connection patterns. Three types of matching matrices are commonly used: input-only, output-only, and combined input-output. Each provides a distinct perspective on connectome structure [76, 85, 86].

The input-only matching matrix measures the similarity of incoming connections. It captures whether two neurons receive input from similar sources, regardless of where they project. This is particularly useful for identifying functionally convergent units–neurons that may play similar integrative roles by sampling from a shared upstream pool. For instance, in layered circuits like the cerebellum or cortex, neurons with similar afferents may participate in common computational motifs [76, 87–89].

In contrast, the output-only matching matrix compares the outgoing connections of neurons. It reflects whether two neurons influence the same or similar downstream targets, regardless of their inputs. This enables the identification of neurons that may serve similar effector functions, such as parallel output channels or broadcast units that drive similar responses across different regions [76, 87–89]..

The combined input-output matching matrix incorporates both afferent and efferent patterns, providing a global measure of connectivity similarity. It is especially valuable for detecting neurons with overall comparable roles in the circuit–both in terms of the signals they receive and the structures they affect. This full-profile comparison helps uncover symmetries, reciprocal motifs, and potential redundancies in the connectome [76, 85, 86].

Together, these three matching matrices offer complementary views on circuit architecture. By analyzing them jointly, one can better identify neuron classes, functional modules, and design principles underlying the organization of biological connectomes.

**Fig. 8:**
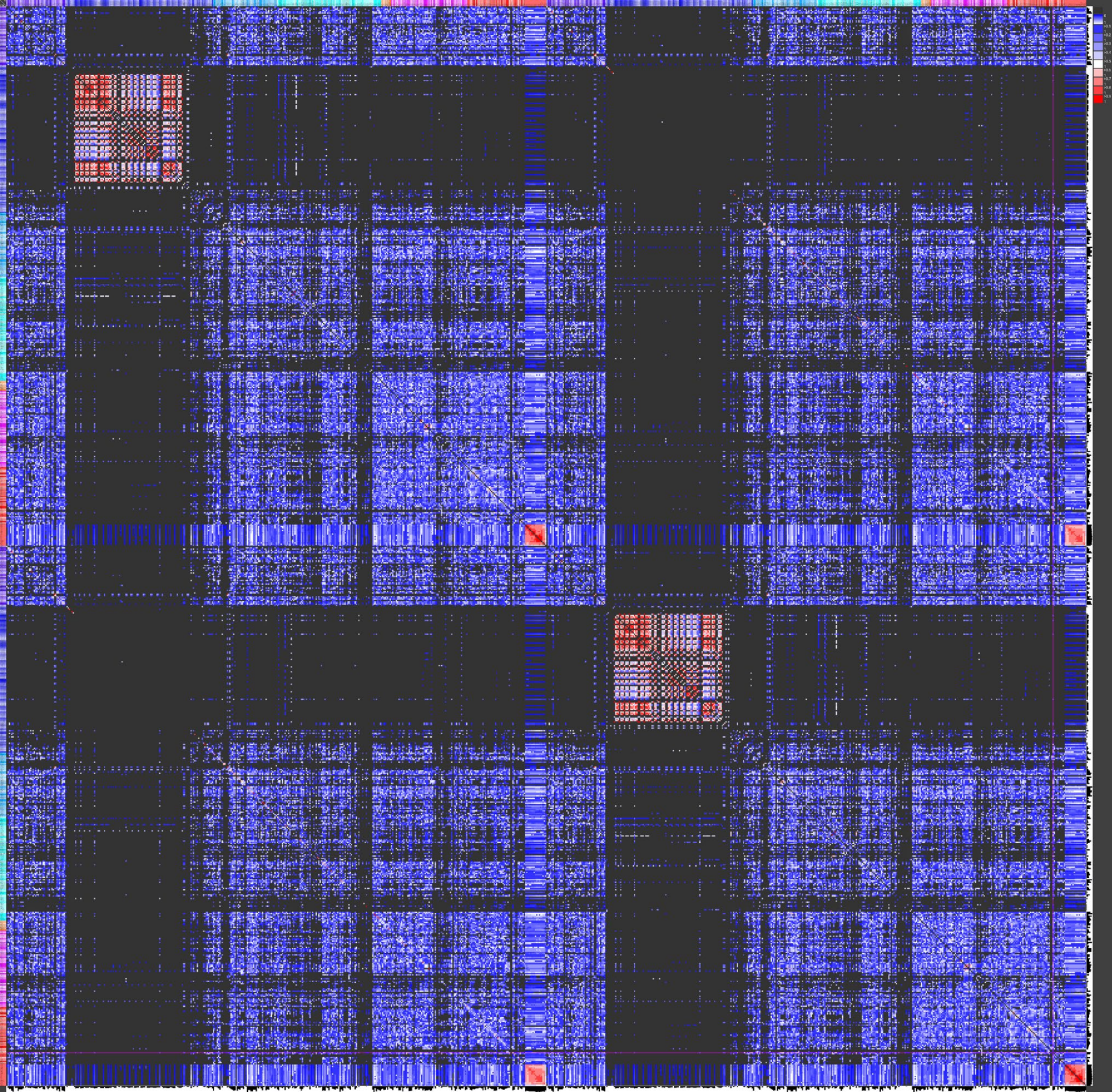
**Connectivity matching index (CMI**$_{all}$**) matrix of the cerebellar connectome**. This symmetric matrix visualizes the CMI_all_ values between all pairs of 862 cerebellar regions, arranged to distinguish left and right hemispheric topographies. The top-left and bottom-right quadrants represent ipsilateral connectivity within the left and right cerebellar hemispheres, respectively. The off-diagonal top-right and bottom-left quadrants illustrate contralateral inter-hemispheric connectivity. The color scale ranges from dark blue (low CMI_all_ values) to bright red (high CMI$_{all}$ values), denoting the degree of similarity between connectivity profiles. Brighter red clusters, especially within ipsilateral quadrants, indicate cerebellar submodules with high mutual coherence, suggesting functional specialization and modular organization. Notably, cerebellar regions tend to exhibit stronger connectivity matching within the same hemisphere, reflecting the intrinsic modular and lateralized structure of the cerebellum

The analysis of the cerebellar connectivity matching matrix (Fig. 8), particularly focused on the region pairs exhibiting maximal $CMI_{all}$ values, reveals notable insights into the intrinsic cerebellar architecture. Several cerebellar subregions, such as those in lobules 6c, 9c, and 10b, consistently reach a $CMI_{all}$ value of 1.0, indicating perfect connectivity profile matching with their respective counterparts. These connections frequently occur between adjacent zones within the same lobule or between functionally related zones across lobules, such as between zones C2 and C3 or D1 and D3. This high degree of coherence suggests a finely tuned modular organization within the cerebellum, where discrete zones operate in tightly integrated functional circuits. Additionally, the presence of bilateral and intra-lobular symmetry, as observed in pairings between right-sided zones of lobules 10a and 10b or between different zones within lobule 9, reinforces the concept of mirrored and redundant connectivity layouts. These characteristics likely contribute to the cerebellum’s efficiency in parallel processing and resilience to localized damage, underscoring its pivotal role in sensorimotor and cognitive integration.

The intracerebellar $CMI_{all}$ matrix, focusing on the left hemispheric cerebellar regions, reveals fine-grained differentiation in connectivity similarity across cerebellar lobules and zones. While certain region pairs display very high $CMI_{all}$ values, reflected by deep red hues, a significant number of matrix cells exhibit lighter tones, such as pale red, white, or even blue, indicating more nuanced variations in connectivity matching. These differences highlight the presence of both tightly and loosely coupled cerebellar substructures.

Notably, regions within the same lobule but across different zones, such as CERL2aC1 and CERL2aC3, tend to exhibit high yet non-identical CMI$_{all}$ values, suggesting shared but distinct connectivity motifs. Similarly, regions like CERL3aD2 and CERL3bC2, although adjacent in topological space, display a mix of high and moderate matching indices, underscoring the influence of both functional and anatomical compartmentalization. These subtle $CMI_{all}$ variations are not random but structured, forming diagonal clusters and patterned groupings in the matrix, particularly in zones C1 to C3 within lobules 2 to 5.

Blue and white regions, indicating low to moderate matching, are often seen between different lobules, such as between lobule 1 (CERL1C1) and lobule 6 (CERL6aC1), signifying reduced overlap in their connectivity profiles. Furthermore, the appearance of sparse or checkerboard-like patterns in some matrix blocks suggests the presence of specialized subnetworks, possibly reflecting functional segmentation even within seemingly homogeneous anatomical labels.

These fine distinctions in intracerebellar $CMI_{all}$ values reveal a complex yet ordered landscape of cerebellar communication, where modularity, gradient transitions, and local specialization coexist. The matrix thus not only confirms the presence of robust modules but also provides a window into the more delicate structure of cerebellar integration, where overlapping connectivity patterns vary in density and directionality across the medial-to-lateral and anterior-to-posterior cerebellar axis.

#### Communicability Analysis

**Fig. 9:**
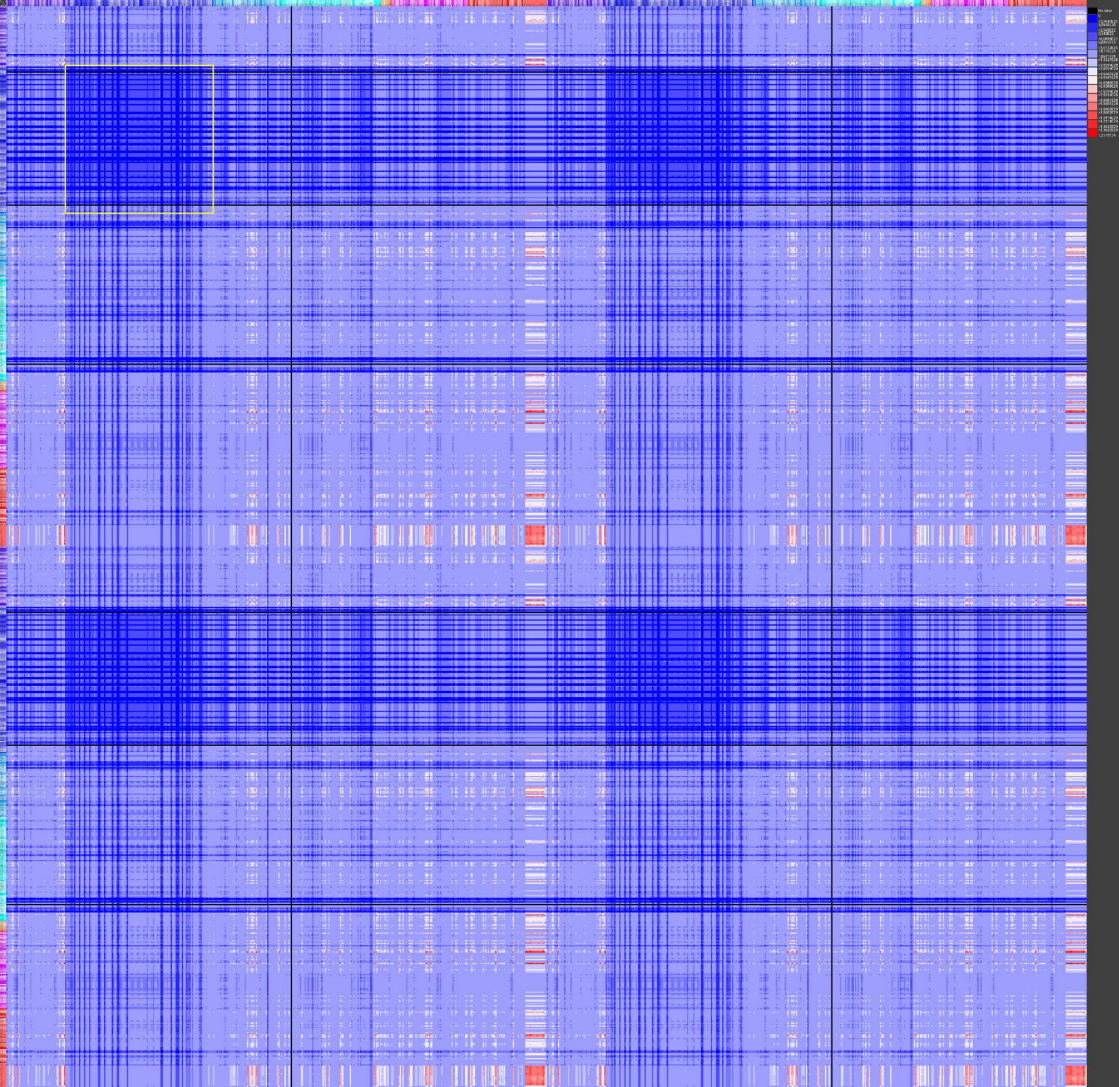
**Color-coded bilateral communicability matrix of the cerebellar connectome**. The matrix displays pairwise communicability values between cerebellar regions (especially dark blue shaded regions with yellow rectangle), reflecting the strength and efficiency of potential information flow across the connectome. Rows and columns represent all regions of the cerebellar connectome from left and right hemispheres. Higher communicability values are encoded in warmer colors (red), indicating densely interconnected and highly traversable region pairs, whereas lower values are shown in cooler tones (blue), suggesting weaker or sparser communicability. The matrix exhibits a prominent block structure, consistent with bilateral symmetry and functional modularity, particularly highlighting strong ipsilateral and homotopic contralateral interactions among cerebellar lobules and nuclei

To investigate the network communicability properties of the brain, we analyzed the maximum communicability output and input values across different neuroanatomical regions. Of particular interest were the ipsilateral cerebellar regions, located in rows 47 to 164 of the communicability dataset (Fig. 9). These values were compared statistically and functionally with all remaining (non-cerebellar) regions of the cerebellar connectome. Technical details of the communicability analysis are explained in Appendix  E.

Let * C * denote the set of ipsilateral cerebellar regions and * N * denote the set of all other regions. The following statistical measures summarize the communicability properties:

$$\begin{aligned} \text {Mean}_{\text {out}}(C)&= 1.33 \times 10^{27},&\text {Std}_{\text {out}}(C)&= 1.13 \times 10^{28}, \\ \text {Mean}_{\text {in}}(C)&= 2.88 \times 10^{27},&\text {Std}_{\text {in}}(C)&= 1.41 \times 10^{28}, \\ \text {Mean}_{\text {out}}(N)&= 2.41 \times 10^{28},&\text {Std}_{\text {out}}(N)&= 3.38 \times 10^{28}, \\ \text {Mean}_{\text {in}}(N)&= 2.68 \times 10^{28},&\text {Std}_{\text {in}}(N)&= 3.79 \times 10^{28}. \end{aligned}$$

The analysis of the maximum pairwise communicabilities within the cerebellar connectome reveals critical insights into the structural and functional integration of cerebellar nuclei and cortical regions. Communicability, as derived from matrix exponential formulations of the adjacency structure (Table 8), quantifies the ease and multiplicity of potential walks between any two nodes in the connectome, reflecting the extent of information propagation and redundancy in the structural architecture.

Among the most prominent patterns observed, cerebellar nuclei–such as the dentate, interposed, and fastigial nuclei–exhibited the highest communicability values with adjacent cerebellar cortical regions, particularly those in lobules VI, VII, and VIII. These elevated values likely mirror the dense projection systems known to exist between deep cerebellar nuclei and specific cortical zones, which are reciprocally linked via the cortico-nuclear loops. The high communicability scores suggest a robust bidirectional integration that facilitates fine-grained timing, predictive motor control, and sensorimotor learning.

Furthermore, intra-cortical cerebellar communicabilities also manifested significant peaks, especially between zones within the same lobule and across mirror-structured hemispheric analogs. For instance, vermal regions of lobule VI and Crus I show strong internal coherence, possibly reflecting their involvement in cognitive and affective regulation beyond sensorimotor functions. These patterns suggest that the cerebellar cortex, while spatially compartmentalized into sagittal zones, is also highly communicative within functionally coherent modules, thereby reinforcing modular but integrated processing.

When comparing the communicability between cerebellar nuclei and distant cortical zones, the values, while generally lower than short-range projections, were nonetheless non-negligible. This indicates the presence of indirect structural routes that may underlie longer-range cerebellar-cortical interactions, including those that support higher-order functions such as language, attention, and working memory. Particularly, the dentate nucleus showed notable communicability with lateral hemispheric regions involved in non-motor tasks, reflecting the anatomical substrate of cerebro-cerebellar loops via the thalamus.

Functionally, these findings underscore the cerebellum’s role not only in localized processing but also in integrative and distributed computation. The communicability structure supports the interpretation that the cerebellum operates as a dynamic relay and modulator within broader brain connectomes, leveraging its internal wiring economy and recursive connectivity. The observed gradients of communicability–from high values in tightly connected cortico-nuclear zones to moderate values in distributed cortical regions–parallel the cerebellum’s functional topography, bridging sensorimotor execution with cognitive-emotional coordination.

The communicability output and input values of cerebellar regions are notably lower than those of non-cerebellar regions. This suggests that cerebellar regions, while still integrated within the brain’s communication network, exhibit reduced direct communicability when compared to cortical and subcortical structures. The significantly lower mean and standard deviation for both incoming and outgoing communicability in cerebellar areas may reflect their specialized role in motor coordination and sensorimotor integration, rather than global network broadcasting or integration functions.

Additionally, the higher standard deviations observed in non-cerebellar regions indicate a broader variability in communicability, consistent with the heterogeneous functional roles of cortical, limbic, and brainstem areas.

These findings underscore the cerebellum’s functionally distinct profile in the brain’s communication topology, suggesting a more modular and possibly hierarchical integration pattern with restricted high-strength communicability channels.

#### Generalized Topological Overlap Matrix (GTOM)

The generalized topological overlap matrix (GTOM) has emerged as a valuable tool for uncovering higher-order topological similarities between brain regions beyond immediate connections. The GTOM extends classical notions of neighborhood overlap and provides insight into the mesoscale architecture of complex neural systems. A more formal description of generalized topology overlap matrix analysis is outlined in Appendix  F.

The GTOM matrix provides a means of assessing the similarity of connectional contexts between regions in the brain, making it especially suitable for identifying shared pathways, redundancies, and potential hubs. In connectomics, it enables to detect modules–groups of regions with similar connectivity profiles–and thus supports modular decomposition of brain architecture.

While the connectivity matching matrix and the generalized topology overlap matrix (GTOM) may appear visually or numerically similar in some contexts, examining both provides complementary insights into the structural and functional architecture of a brain network. The connectivity matching matrix (CMM) (see above) quantifies direct connectivity similarity between pairs of nodes, typically by comparing how similarly they connect to other nodes, for example using cosine similarity or the Jaccard index. The generalized topology overlap matrix (GTOM), in contrast, incorporates indirect and higher-order topological relationships, such as how many neighbors two nodes share at various distances, not just among immediate neighbors. Even when visually similar, the GTOM is sensitive to hierarchical or modular structure, which may not be captured by basic connectivity matching.

The CMM often highlights redundancy or functional similarity, typically identifying nodes with similar roles. GTOM is better suited to revealing community structure and topological roles, such as hubs, bridges, or peripheral nodes. As a result, GTOM can differentiate between nodes that look similar in terms of direct connectivity but play very different roles within the broader network topology.

GTOM is also frequently applied in module or cluster detection tasks because it considers shared connections, path-based overlaps, and indirect influences. This allows it to provide a more nuanced picture of functional neighborhoods in the brain compared to CMM, which might overestimate similarity when nodes reside in highly connected areas, such as rich clubs.

Furthermore, GTOM tends to be more robust to noise or incomplete data, as it aggregates information over multiple connection paths. In contrast, the CMM can be distorted by small errors in local connectivity, making GTOM a more reliable descriptor in practical applications.

In the context of neurobiology, GTOM correlates more strongly with developmental patterns, plasticity, and disease propagation. Meanwhile, the CMM reflects functional similarity primarily at a local scale. Although both matrices offer useful insights, GTOM is often more aligned with long-range or evolutionarily preserved network motifs and architectures.

Even when the connectivity matching matrix and GTOM appear similar, they represent different levels of abstraction. The CMM captures local, connection-based similarity, whereas GTOM reflects global, path-based topological overlap. Their similarity may indicate a strong underlying modularity in the network. However, to fully understand the network’s organizational principles–especially in complex systems like the brain–it is essential to analyze both perspectives.

By considering paths of increasing length, the GTOM reveals overlapping neighborhoods that may not be visible through direct adjacency. This is particularly relevant in the study of integrative regions, such as cortical hubs and cerebellar nuclei, which exhibit broad communication profiles. A high GTOM score between two regions suggests they are embedded in comparable connectome environments, possibly contributing to shared or complementary functions.

GTOM has been applied in both structural and functional connectome studies to detect modules, assess disease-related topological disintegration, and compare connectome organization across subjects. It is especially useful in cerebellar connectomics, where modular and bilateral organization patterns are prominent. The matrix helps to identify topologically coherent clusters and offers a refined lens for understanding how information is distributed and processed in cerebellar circuits.

The cerebellar submatrix shows a higher average pairwise topological overlap compared to the full GTOM matrix Fig. 11. Specifically, the mean GTOM value for cerebellar-cerebellar connections is approximately 0.176 (*σ * 0.272), while the global mean across all regions is approximately 0.151 (*σ * 0.230). This suggests that cerebellar regions, as a group, exhibit relatively stronger local topological clustering than the brain network as a whole. A higher GTOM value between two nodes indicates they share many topological features, particularly overlapping neighbors across multiple orders. The elevated mean GTOM in the cerebellum suggests a greater internal consistency and modularity within the cerebellar network as well as shared connectivity patterns that may support the cerebellum’s role in coordinating and integrating sensorimotor information. Furthermore, this indicates a potential for redundant or robust pathways, possibly contributing to fault-tolerance in cerebellar processing. The standard deviation is also slightly higher in the cerebellar block, reflecting greater heterogeneity in how cerebellar regions participate in shared topological neighborhoods – some regions may act as central hubs, while others remain more peripheral.

**Fig. 10:**
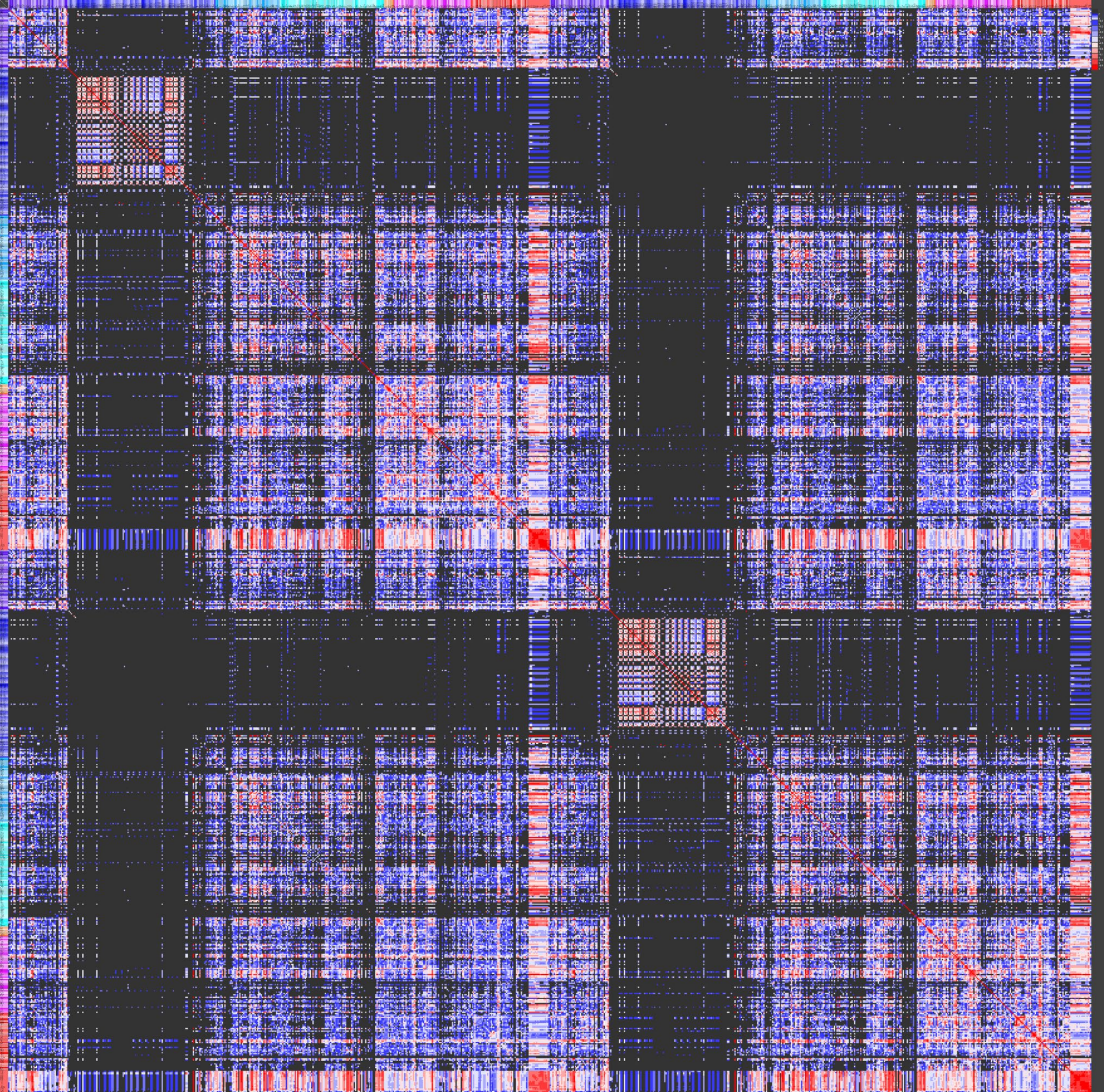
**Generalized Topological Overlap Matrix (GTOM) of the cerebellar connectome**. Color-codes represent the degree of topological similarity between brain regions. Each element in the matrix reflects the extent of shared neighbors between a pair of regions, thereby quantifying their connectome proximity beyond direct connections. Higher GTOM values, indicated by warmer colors (red), imply a stronger overlap in connectivity profiles, suggesting possible functional coordination or shared roles within a connectome module. Cooler colors (blue to black) correspond to low topological overlap and likely functional separation. The highlighted yellow rectangle marks cerebellar structures, including the cerebellar cortex and deep cerebellar nuclei, which exhibit a high degree of internal topological coherence. This localized clustering indicates that cerebellar subregions are not only densely interconnected but also embedded within distinct topological modules, possibly reflecting their integrative roles in sensorimotor and cognitive processing

The hierarchical clustering dendrogram of cerebellar regions based on GTOM similarity is shown in Fig. 10. Each branch reflects the degree of topological overlap among regions, with closely clustered branches indicating strong similarity in network embedding. Some cerebellar regions cluster tightly, suggesting shared roles or redundant connectivity patterns. The variation in linkage distances indicates heterogeneity–some regions are central to cerebellar modules, others more topologically distinct.

**Fig. 11:**
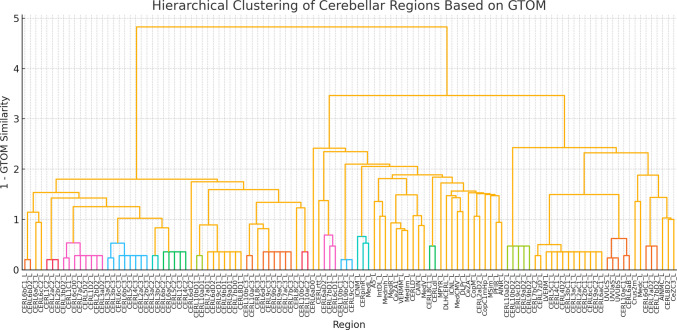
**Hierarchical clustering of cerebellar regions based on generalized topology overlap matrix (GTOM)**. The dendrogram illustrates the clustering structure of ipsilateral cerebellar regions (rows and columns 48 to 165 of the GTOM matrix) using hierarchical agglomerative clustering. The input similarity matrix was derived from the pairwise GTOM values, which quantify higher-order topological overlap between neuroanatomical regions. To compute distances, the GTOM values were converted to dissimilarities by subtracting each value from 1, resulting in a distance metric defined as $d_{ij} = 1 - \text {GTOM}_{ij}$. The linkage method applied was *average linkage* (UPGMA), which merges clusters based on the mean distance between all pairs of regions across clusters. The hierarchy of clusters reveals modular organization within the cerebellar network, with shorter vertical linkages indicating higher topological similarity. Leaf labels correspond to region abbreviations, and the vertical height of each merge reflects the distance threshold at which clusters are joined

To assess the community structure of the cerebellar network, we performed a modularity analysis based on hierarchical clustering derived from the generalized topology overlap matrix (GTOM) [90–95]. First, the pairwise GTOM similarity values among cerebellar regions (rows and columns 48 to 165 of the full GTOM matrix) were transformed into a distance matrix using the transformation $d_{ij} = 1 - \text {GTOM}_{ij}$. This dissimilarity matrix served as input to an agglomerative clustering algorithm using average linkage. The number of clusters was empirically set to six, reflecting an intermediate resolution of the network’s modular architecture.

To compute modularity, we constructed an undirected weighted graph where nodes represented cerebellar regions and edges were defined by thresholding the GTOM similarity matrix at a value of 0.2. That is, only edges with $\text {GTOM}_{ij} \ge 0.2$ were retained, emphasizing stronger topological overlap. The cluster labels obtained from the hierarchical clustering were then used as predefined community assignments. The modularity score was computed using the standard definition:

$$ Q = \frac{1}{2m} \sum _{i,j} \left[ A_{ij} - \frac{k_i k_j}{2m} \right] \delta (c_i, c_j) $$

where $A_{ij}$ is the edge weight between nodes * i * and * j *, $k_i$ and $k_j$ are the respective node strengths (weighted degrees), * m * is the total edge weight in the network, and $\delta (c_i, c_j)$ is the Kronecker delta which equals 1 if nodes * i * and * j * belong to the same cluster. The resulting Newman modularity value of * Q = 0.097 * suggests a modest degree of modular organization within the cerebellar network. This reflects that while the identified clusters exhibit some degree of internal cohesion, the overall separation between modules is not pronounced, possibly indicating overlapping functional domains or a continuous gradient of connectivity patterns in the cerebellum.

The cerebellar regions were successfully clustered into 6 groups using agglomerative clustering based on their GTOM-derived topological similarity. The computed modularity score for this clustering is 0.097, indicating a modest level of community structure – that is, the clusters are somewhat more densely interconnected internally than externally, though not strongly so.

The two-dimensional t-distributed Stochastic Neighbor Embedding (t-SNE) projection provides a low-dimensional embedding of the high-dimensional generalized topology overlap matrix (GTOM), which captures indirect and higher-order connectivity similarities between neuroanatomical regions. Each point in the plot represents a single brain region, and spatial proximity in the embedded space reflects the degree of topological similarity between regions as encoded in the GTOM.

**Fig. 12:**
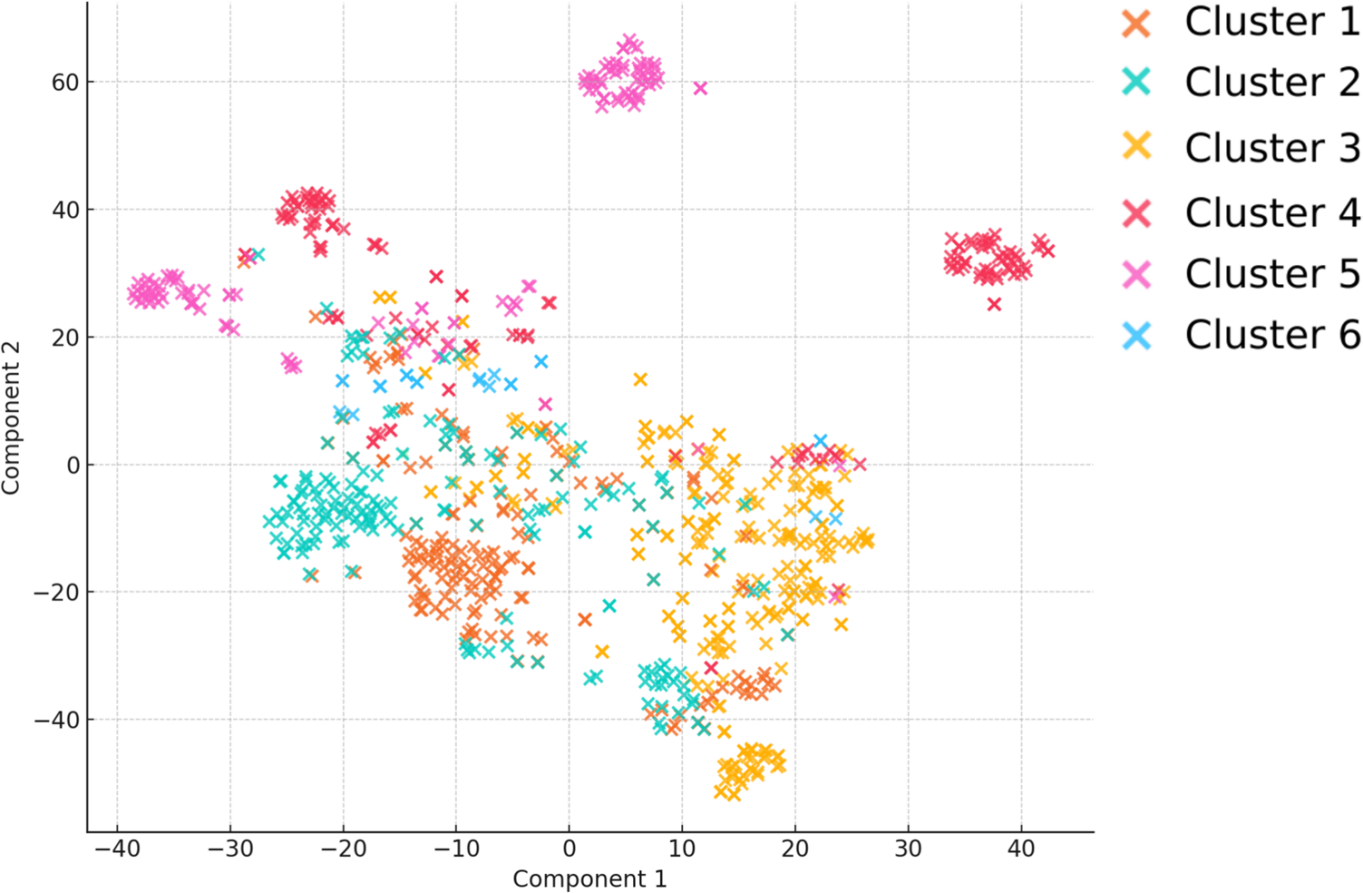
**t-SNE projection of the GTOM matrix with Louvain cluster assignments and cerebellar region highlighting.** This two-dimensional t-distributed Stochastic Neighbor Embedding (t-SNE) visualization is based on the original generalized topology overlap matrix (GTOM), which captures higher-order topological similarity between neuroanatomical regions. Each point in the plot corresponds to a single region, projected into a low-dimensional space such that regions with similar network topology are positioned closer together. Colors indicate membership in one of six consensus clusters identified through Louvain community detection. The plot reveals spatially coherent and partially distinct clusters, reflecting the modular structure of the connectome as captured by the GTOM. Cerebellar regions appear as a concentrated subset within the embedding, supporting their role as a topologically cohesive and structurally distinctive subnetwork within the whole-brain architecture

The t-SNE was computed using a perplexity of 30 and standard preprocessing by feature-wise normalization. To interpret the resulting structure in terms of modular organization, points were color-coded based on their assignment to six consensus Louvain clusters (see above) (Fig. 12), previously identified through community detection on the same GTOM. In addition, cerebellar regions–including those with anatomical labels indicating cerebellar lobules, deep cerebellar nuclei, and vestibular-cerebellar junctions–were visually emphasized using black-edged markers.

The projection reveals distinct groupings of regions that largely correspond to the detected Louvain modules, suggesting that the GTOM preserves modular topology in the reduced space. Cerebellar regions form relatively compact and partially segregated clusters, indicating that their network embedding is both coherent and topologically distinct from many forebrain and brainstem regions. The continuity and partial overlap among clusters reflect gradients of topological similarity, especially across multisensory integration areas and limbic subcortical structures. The presence of spatially isolated nodes with "unassigned" labels also suggests potential outlier regions or transitional zones between modules.

Overall, this projection confirms that the modular organization derived from Louvain clustering is supported by the intrinsic structure of the GTOM, and that cerebellar regions form a functionally and topologically meaningful subnetwork within the broader brain connectome.

#### Vulnerability Analysis

Vulnerability analysis plays a central role in understanding the structural and functional robustness of the cerebellar connectome [96]. The cerebellum, traditionally associated with motor coordination, has increasingly been recognized as a key structure involved in a range of cognitive and affective functions. This expanded role suggests that its network organization may be optimized for both efficiency and resilience, making it essential to understand how its connectivity responds to targeted disruptions [97].

By systematically removing connections in a principled manner—for example, based on edge significance derived from anatomical, statistical, or functional measures—vulnerability analysis reveals the extent to which specific subsets of connections contribute to maintaining critical network properties. A particularly informative metric in this context is the clustering coefficient [6, 98], which captures the local density of interconnected regions and reflects the network’s modularity and capacity for localized processing.

Applied to the cerebellar connectome, vulnerability analysis can uncover whether the network relies heavily on a small set of highly significant connections, or whether it is organized more redundantly and robustly. A rapid decline in the clustering coefficient upon removal of high-significance edges indicates a fragile, non-redundant topology, suggesting that certain connections are essential for maintaining functional integrity. Conversely, a stable profile under such perturbation implies robustness, possibly due to distributed or degenerate connectivity patterns.

Moreover, comparing the empirical vulnerability profile to those derived from surrogate networks—which preserve low-level features such as degree distribution but randomize higher-order structure—enables the identification of non-random, functionally optimized architectural features. In the case of the cerebellum, such analysis may reveal whether its network structure is the result of evolutionary pressure favoring specialized integration, modularity, or resilience.

In the context of a weighted brain network (e.g., the cerebellar connectome), vulnerability analysis refers to the systematic evaluation of a network’s robustness or fragility by progressively removing edges according to a predefined metric (e.g., edge significance), and measuring a network property (e.g., average clustering coefficient) after each removal step. The technical details are explained in more detail in Appendix H.

The vulnerability diagram (Fig.) 13) shows four key sets of curves plotted over the number of removed edges (x-axis) vs. the mean clustering coefficient (y-axis). Green curve (empirical cerebellar connectome, high *→ * low significance removal) displays a sharp drop with irregular oscillations. This indicates that the network becomes significantly less clustered when highly significant edges are removed first. The erratic behavior suggests nonlinear vulnerability and critical dependencies on certain high-significance edges. The blue curve (empirical cerebellar connectome, removal of edges with low *→ * high significance) shows that the clustering coefficient decreases slowly and evenly, suggesting that edges with low significance have less structural influence on clustering in early stages. The pink curves (30 surrogate rewiring networks, high *→ * low removal) are the surrogate equivalents of the green curve. They decline more gradually and smoothly, lacking the instability seen in the empirical network. This suggests that empirical significance is non-random and functionally relevant. The cyan curves (30 surrogate rewiring networks, low *→ * high removal) are analogous to the blue curve for the null models. These also show a gentle decline, confirming that low-significance edges are not critical for clustering in either empirical or surrogate cases.

The empirical cerebellar connectome is highly vulnerable to the removal of edges with high significance. This is evident from the rapid and unstable drop in clustering (green curve), not mirrored by surrogate networks. This asymmetry (high *→ * low vs. low *→ * high) in the empirical network indicates that the distribution of edge importance is non-uniform, with certain edges acting as structural “hubs” for local clustering. The surrogate networks, which preserve some topological constraints but randomize edge patterns, show more gradual and symmetrical declines. This supports the interpretation that the cerebellar connectome is topologically optimized with respect to certain high-significance edges.

**Fig. 13:**
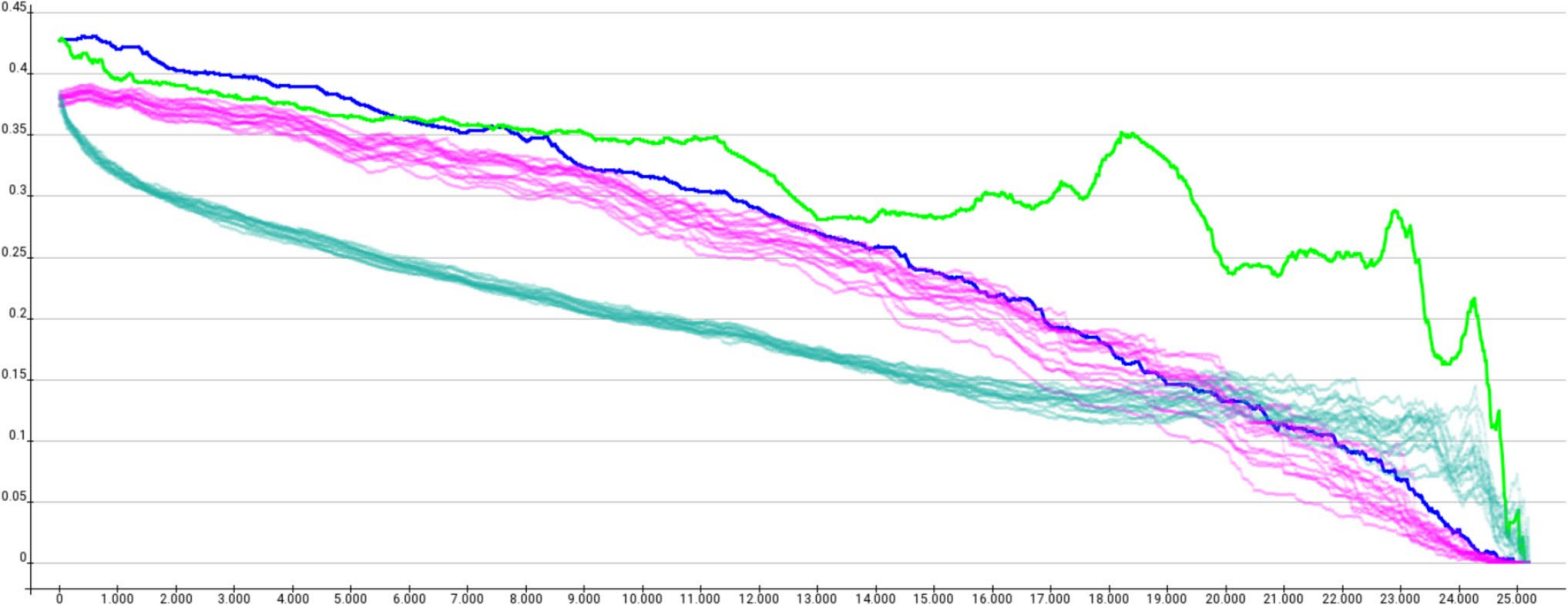
**Vulnerability analysis of the empirical cerebellar connectome and rewired surrogates**. The average clustering coefficient is plotted against the number of removed edges under four different conditions: (1) – empirical cerebellar connectome, edges removedfrom high to low significance; (2)  – empirical cerebellar connectome, edgesremoved from low to high significance; (3)  – 30 rewiring surrogate networks,edges removed from high to low significance; (4)  – 30 rewiring surrogatenetworks, edges removed from low to high significance. High-to-low significance removal in the empirical network causes a steep, irregular drop in clustering, indicating topological fragility and the crucial role of significant edges. In contrast, both removal directions in surrogate networks show smoother and more gradual declines, suggesting topological redundancy in randomized architectures. This highlights the functional relevance and nonrandom structure of the empirical cerebellar connectome

Summary of connectome properties to facilitate interpretation of the extensive network analyses presented above, the principal findings across analytical domains are summarized in Table 4. Overall, the cerebellar connectome exhibits a combination of sparse connectivity, strong hub dominance, modular organization, and non-random motif structure. Together, these features indicate a highly organized network that balances local specialization with global integration, consistent with known principles of biological brain networks.

**Table 4:** Summary of key findings across analytical domains of the cerebellar connectome

Domain	Key metric(s)	Main finding	Interpretation
Global topology	Density, path length	Sparse but efficient	Consistent with biological networks
Modularity	Modularity, clustering	Moderate modular structure	Functional specialization
Hubs	Centrality, central point dominance (CPD)	Strong hub dominance	Hierarchical organization
Motifs	Motif enrichment	Non-random motifs	Structural constraints
Vulnerability	Edge removal analysis	High sensitivity to key edges	Non-random architecture

## Discussion

This study provides a comprehensive connectomic analysis of the cerebellar network, revealing several key organizational principles. Across multiple analytical domains, the cerebellar connectome was characterized by sparse but efficient global topology, pronounced hub dominance, moderate modular organization, and a non-random motif structure. Together, these features indicate a highly structured network that balances local specialization with global integration. In addition, the network exhibits strong sensitivity to the removal of high-significance edges, suggesting a non-uniform distribution of structural importance and the presence of critical connectivity backbones.

These findings extend current understanding of cerebellar organization by demonstrating that its connectivity follows general principles observed in complex biological networks, while also exhibiting distinct features related to modular processing, bilateral symmetry, and hierarchical integration. The present study is grounded in the growing recognition that the cerebellum plays a far more diverse role in brain function than traditionally assumed. As outlined in the introduction, the motivation for analyzing the cerebellar connectome stems from both a scientific gap and a conceptual opportunity: despite increasing evidence linking the cerebellum to cognitive, affective, and autonomic domains, its connectivity remains relatively underexplored compared to the cerebral cortex. The structural complexity of the cerebellum–marked by modularity, laminar architecture, and intricate afferent-efferent loops–makes it a prime candidate for connectome-based analysis [42, 99–101].

Using the laboratory rat as a model offers a pragmatic and biologically valid approach, given its well-mapped neuroanatomy, availability of high-resolution tract-tracing data, and broad relevance in translational research. Furthermore, the focus on partial connectomics, particularly the intrinsic and extrinsic subdivisions of cerebellar connectivity, is a reasonable methodological strategy in light of incomplete data coverage and the need for tractable network models. The inclusion of lateralization (left, right, and contralateral connectivity) adds a valuable dimension, recognizing that hemispheric asymmetries may underlie functional specialization and compensatory mechanisms following injury [59, 102–106].

Critically, however, the motivation rests on several assumptions that should be addressed in future work. First, while rodent models are highly informative, the generalization of cerebellar connectivity patterns to higher mammals or humans should be made cautiously, especially in cognitive domains. Second, the distinction between intrinsic and extrinsic connectivity, while anatomically grounded, may oversimplify dynamic and reciprocal interactions across networks, especially in closed-loop systems. Third, the reliance on static structural connectivity may miss key aspects of cerebellar function that emerge only through dynamic, context-dependent interactions [51, 100, 107–110].

Finally, while graph theory and dynamic systems analysis offer powerful frameworks for formalizing cerebellar organization, the interpretability of metrics such as clustering or hubness in biological terms remains an open challenge. The predictive utility of these measures for behavior, learning, or disease modeling will ultimately determine the translational relevance of cerebellar connectomics.

The rationale for cerebellar connectome analysis is both timely and well justified, but future directions should seek to expand the scope beyond static models, incorporate cross-species comparisons, and integrate multimodal data to fully realize the promise of cerebellar systems neuroscience.

### Methodological Considerations and Limitations

In this study, the cerebellar connectome was extracted and analyzed from a meta-analytic, large-scale database of over 7,800 tract-tracing studies, providing a richly annotated, hierarchically structured, and directionally weighted network model. The neuroVIISAS framework served as the primary tool for query, modeling, and visualization, enabling integration of anatomical ontologies, graph-theoretical analysis, and simulation-ready data export. The cerebellar subnetwork was isolated through targeted hierarchical expansion and systematic inclusion of input/output neighbors, followed by topological filtering to retain only regions with at least one afferent and one efferent connection.

While this approach allows for detailed and scalable modeling of the cerebellar architecture, several methodological limitations merit consideration. First, despite the comprehensiveness of the underlying dataset, it remains subject to the heterogeneity of the included primary studies–differing in species, experimental protocols, tracer types, and anatomical resolution. These factors may introduce biases or artifacts in the network topology, especially in sparsely studied subregions. Second, the tract-tracing data, though directionally resolved, do not directly convey synaptic specificity, laminar origin, or the functional efficacy of connections. Consequently, the resulting network remains a structural approximation, lacking detailed microcircuitry information that would be required for fully realistic simulations.

Moreover, the hierarchical region selection and pruning steps, while necessary for graph-theoretical consistency, involve decisions (e.g., leaf expansion depth, neighbor inclusion) that may affect the final network composition and connectivity density. Although the removal of unconnected nodes ensures a fully connected subgraph, it may exclude biologically meaningful but weakly connected areas, thus potentially underestimating functional integration.

Finally, the neuroVIISAS framework, although well suited for anatomical data integration, currently offers limited integration of multimodal data such as electrophysiology or gene expression in the basic version used here. Future work should aim to extend the methodological pipeline to include such modalities and incorporate probabilistic or dynamic connectivity estimates.

Despite these limitations, the methodological strategy adopted here offers a reproducible and systematically derived cerebellar connectome that serves as a robust substrate for structural and computational analyses.

### Local Cerebellar Circuit Reconstruction

The results of this study demonstrate that local cerebellar microcircuit motifs can be reliably extracted and represented from a high-resolution, macroconnectome-derived dataset. The reconstructed adjacency matrix and hierarchical diagram (Figs. 2 and 3) successfully recapitulate canonical cerebellar pathways, including mossy and climbing fiber afferents, parallel fiber networks, interneuron-mediated inhibition, and deep cerebellar nuclear outputs. Moreover, the inclusion of lesser-studied elements such as Lugaro and unipolar brush neurons affirms the capacity of the connectome framework to represent complex local circuit dynamics and integrate both excitatory and inhibitory subnetworks [61, 111–114].

From a critical perspective, however, several caveats must be acknowledged. First, although the macroconnectome-based approach allows for topological inferences at mesoscale resolution, it inevitably abstracts away from the synaptic, laminar, and microcolumnar detail that defines cerebellar computation at the cellular level. The mapping of directionality and neurotransmitter identity relies on established literature and inferred connectivity rules rather than direct evidence from ultrastructural or single-cell tracing data.

Second, while the observed lateralization patterns and ipsilateral versus contralateral projections (e.g., uncrossed mossy fibers, crossed climbing fibers) align with known neuroanatomy, the current model assumes spatial homogeneity within regions. This may overlook local gradients or spatial clustering of functional submodules, particularly in lobule-specific or domain-specific circuits such as those supporting vestibular, oculomotor, or cognitive functions.

Third, the inclusion of rare or sparsely distributed cell types, such as Lugaro and Brush neurons, highlights the modeling potential of the framework but also raises questions about data coverage and confidence. Their functional roles are well characterized in selected cerebellar domains, yet their integration into a general connectome may require more localized data to avoid overgeneralization.

Lastly, while the proposed expansion toward a mesoscale cerebellar connectome linking midbrain, diencephalon, and cortex is promising, it will necessitate careful reconciliation of inter-regional data heterogeneity and standardized ontological frameworks to preserve biological plausibility across scales.

In conclusion, the extraction of cerebellar microcircuits from a macroconnectome provides a compelling proof-of-concept for hierarchical, functionally annotated connectomics. Future work should aim to integrate higher-resolution, cell-specific data, include dynamic modeling components, and validate inferred motifs experimentally to enhance the explanatory and predictive power of cerebellar network models.

### Mesoscale Cerebellar Connectome Analysis

The mesoscale analysis of the bilateral cerebellar connectome presented a high-resolution, graph-theoretic profile of the cerebellar system, revealing a densely integrated but sparsely connected architecture. The empirical network comprised 862 nodes and over 21,000 directed edges, including contralateral and recurrent motifs, indicative of a complex and biologically plausible topology. Notable features included a high degree of reciprocity, modular organization, moderate clustering, and efficiency metrics characteristic of small-world and scale-free networks. Importantly, model comparisons revealed that no single generative algorithm fully reproduced all observed properties, although the modified OHO and KE small-world models approximated selected features such as clustering, modularity, and cyclicity.

Critically, while the network analysis provides valuable quantitative descriptors of cerebellar architecture, several limitations should be addressed. First, the mesoscale model abstracts from underlying microcircuit details and omits cell-type specificity. This may mask functional heterogeneity across regions, especially within cerebellar subnuclei or lobules involved in specialized tasks such as vestibular processing or eye movement control [109, 111, 112, 115, 116].

Second, the inference of functional organization from structural topology remains indirect. While global measures like clustering coefficient, path length, or modularity suggest efficiency and compartmentalization, they do not directly capture the dynamic behavior of neural circuits under physiological conditions. The incorporation of simulation-based validation–e.g., using spiking models or dynamic causal modeling–could help bridge this gap.

Third, although the comparison with synthetic network models is methodologically rigorous and statistically robust, it assumes that the biological connectome should be well-approximated by abstract graph algorithms. In reality, developmental, metabolic, and spatial constraints introduce constraints that may not be reproducible via purely statistical models. The excessive centrality values observed (e.g., extreme CPD) might reflect true hub dominance, but they could also indicate limitations in the modeling of weight distributions or resolution of bidirectional links [26, 109, 117–119].

Lastly, while the connectome exhibits scale-free and small-world tendencies, these classifications are sensitive to thresholding, edge weight treatment, and network resolution. Further multi-scale validation–potentially involving cross-species comparisons or longitudinal data–would strengthen the generalizability of these observations.

In summary, the mesoscale cerebellar connectome analysis demonstrates the rich structural and organizational complexity of cerebellar connectivity. While it provides a strong foundation for systems-level modeling and hypothesis generation, future work should aim to integrate multimodal and dynamic data to refine the biological realism and interpretability of the network models.

### Local Connectome Analysis

The local connectome analysis provided a detailed, multimetric characterization of nodal properties within the cerebellar connectome, highlighting both global influence and regional specialization. By reducing an initial set of 51 graph-theoretical metrics to a strategically selected subset of 11 core parameters, the analysis achieved a balance between interpretability, neurobiological relevance, and statistical coverage. Key findings include the identification of the *pedunculopontine tegmental nucleus, pontine reticular nucleus*, and *parabrachial nucleus* as structurally central and functionally critical nodes, characterized by high degree centrality, radiality, modular participation, and vulnerability indices [26, 61, 109, 112, 120, 121].

The approach offers several strengths. The grouping of parameters into coherent functional domains–such as topology, modularity, spatial embedding, controllability, and vulnerability–enables a multidimensional understanding of each region’s role. Furthermore, the integration of both intrinsic (e.g., clustering coefficient, hubness) and extrinsic (e.g., participation coefficient, Sig) metrics allows for nuanced functional inferences across cerebellar subregions.

However, some limitations must be acknowledged. First, while the analysis accounts for bilateral symmetry and modular gradients, it does not explicitly model temporal dynamics or plasticity, which are essential for understanding cerebellar adaptation and learning. Second, the reliance on structural topology may obscure cell-type specific effects or neurotransmitter diversity–factors known to shape cerebellar computation. Third, although the average rank offers a useful summary score, it may flatten meaningful heterogeneity across parameter categories, potentially obscuring specialized nodes that excel in only one or two domains.

Moreover, the absence of a rich-club or knotty center, as indicated by uniformly low Knot values, raises interesting questions about the organizational logic of the cerebellum. While this may reflect an optimized modular-parallel architecture, it might also suggest that certain integrative features are delegated to extracerebellar regions or emerge dynamically through activity-dependent processes. Additionally, the interpretation of modal controllability (MC) is constrained by its structural basis, and future studies incorporating empirical neural dynamics will be essential to validate its predictions.

In summary, the local connectome analysis provides compelling evidence for a functionally differentiated cerebellar architecture, where specific brainstem and nuclear regions act as integrative hubs, while cortical regions maintain localized processing roles. The observed gradients of modularity, vulnerability, and spatial embedding reinforce theoretical models of cerebellar hierarchy and specialization. Future work should extend these insights through longitudinal, multimodal, or simulation-based approaches to fully elucidate the operational principles of cerebellar network function.

### Motif Analysis

The motif analysis of the cerebellar connectome revealed distinct patterns of local circuit motifs that significantly deviate from random graph models. By comparing empirical motif frequencies to distributions obtained from 1000 rewired surrogate networks, the analysis identified a subset of triadic motifs (e.g., 3-04, 3-06, 3-08, 3-11, 3-13) that were highly overrepresented, as indicated by large positive *z*-scores and *p*-values *<0.001*. These motifs likely reflect functionally and developmentally constrained substructures, rather than arising from general topological rules. Conversely, several feedforward-like motifs (e.g., 3-01 through 3-03) were common in both empirical and rewired networks, suggesting they represent baseline architectural features rather than cerebellum-specific motifs [122–126].

Critically, the findings support the hypothesis that the cerebellar connectome is not only non-random, but also enriched in specific recurrent and reciprocal microcircuits. Motifs such as 3-11 and 3-13, which involve bidirectional and cyclic interactions, may underpin core cerebellar computations such as predictive coding, feedback control, and error correction. Their statistical overrepresentation suggests that cerebellar architecture is tuned for dynamic coordination and stability.

However, some caveats merit attention. First, motif analysis is inherently limited to small subgraph configurations and may not capture larger-scale patterns such as modularity or hierarchy. Second, the analysis is based solely on structural topology without incorporating synaptic weights, laminar origin, or dynamic interactions, all of which influence functional relevance. Third, while statistically significant, the functional interpretation of motifs remains speculative without complementary physiological or modeling data.

Moreover, the sensitivity of motif counts to graph density, edge directionality, and node labeling must be carefully considered. Although the analysis controls for overall degree distribution via rewiring, subtler spatial or developmental constraints may still confound interpretations. For example, motifs rare in surrogate models may not necessarily reflect optimization but instead arise from biological constraints such as projection fields or lineage.

In conclusion, the motif analysis substantiates the presence of non-random and potentially functionally meaningful microstructures in the cerebellar connectome. It complements the broader topological analyses by offering insight into localized structural building blocks. Future studies should aim to validate these motifs experimentally and explore their role in shaping dynamic cerebellar function.

### Modularity and Functional Subsystems in the Cerebellar Connectome

The application of community detection, specifically via the Louvain modularity algorithm, yielded six consensus clusters in the cerebellar connectome that exhibit coherent structural and functional themes. These clusters transcend traditional anatomical boundaries and suggest the presence of functionally meaningful modules–ranging from bilateral cortical-limbic systems and segmental relay zones to cerebellar-brainstem integration and brainstem-autonomic hubs [100, 109, 127–129].

This modular decomposition provides several important insights. First, the detected communities reflect both hemispheric symmetry and functional differentiation. For instance, Clusters 1 and 2 capture homologous sensorimotor-limbic modules in the left and right hemispheres, respectively. This bilateral organization supports cerebellar roles in symmetric motor control and sensory integration. Second, the presence of distinct modules for thalamic relay (Cluster 3), multimodal sensory integration (Cluster 4), and proprioceptive-cerebellar feedback (Cluster 5) emphasizes the cerebellum’s diverse engagement in sensorimotor and cognitive functions. Notably, Cluster 6 includes deep brainstem structures involved in motor-autonomic coordination, pointing to integrative hubs at the interface of visceral and motor regulation.

From a methodological perspective, the use of consensus clustering stabilized the inherent variability in community detection and enhanced the reliability of assignments. The hierarchical nesting of regions preserved anatomical context and allowed fine-grained interpretation of functional subsystems.

Critically, however, several limitations merit consideration. First, the community detection approach is sensitive to input matrix type (e.g., raw connectivity vs. GTOM), resolution parameters, and the stochastic nature of the algorithm itself. Although consensus clustering mitigates these factors, some degree of uncertainty remains in module delineation. Second, community detection is inherently a data-driven method–it reveals modular organization based on connectivity patterns but does not inherently inform on causal or dynamic interactions between modules. Complementary functional data (e.g., fMRI, electrophysiology) would strengthen claims about the behavioral or computational roles of these modules.

Moreover, while the Louvain method captures non-overlapping modules efficiently, it does not explicitly account for overlapping communities or hierarchical layers beyond a single level. Given the known nested architecture of cerebellar and brainstem circuits, future work could benefit from multi-resolution or overlapping community detection methods.

In summary, the modular architecture uncovered through community detection supports a functionally differentiated yet integrative model of the cerebellar connectome. It provides a compelling basis for relating network topology to domain-specific and cross-domain cerebellar functions and may serve as a valuable framework for exploring alterations in connectivity in neurological and psychiatric disorders.

### Connectivity Matching and Intracerebellar Circuit Similarity

Connectivity matching analysis offers a powerful tool for quantifying structural similarity between cerebellar regions based on their afferent and efferent patterns. In the current study, the combined connectivity matching index (CMI$_{all}$) revealed that multiple cerebellar subregions–particularly within lobules 6c, 9c, and 10b–exhibited high or perfect mutual similarity, often approaching a CMI$_{all}$ value of 1.0. These findings highlight the existence of tightly integrated microzones and repeated circuit motifs that support the cerebellum’s modular and hierarchical architecture [55, 100, 111, 130, 131].

From a structural standpoint, the strong ipsilateral matching–especially within individual lobules or between adjacent zones–reinforces the notion of intra-hemispheric functional compartmentalization. Symmetry between paired zones, such as C2/C3 or D1/D3, further suggests that homologous or complementary processing units are mirrored across the cerebellar cortex. Importantly, these repeating motifs are not uniformly distributed; while some regions show high degrees of similarity, others exhibit moderate or low CMI$_{all}$ values, indicating a mixture of conserved and specialized circuitry.

This nuanced patterning implies that cerebellar modules operate within a gradient of connectivity coherence: some subregions exhibit stereotyped communication profiles, while others participate in more diverse or context-dependent interactions. The diagonal clustering and checkerboard-like distributions observed in the CMI$_{all}$ matrix underscore the presence of both tightly coupled circuits and functional segregation, which may support distinct temporal or sensorimotor computations across lobules.

Critically, while high connectivity matching suggests redundancy and resilience, it also raises the possibility of overestimating functional equivalence. Perfect profile similarity does not necessarily imply identical physiological roles or dynamic properties. Furthermore, the approach assumes static connectivity, neglecting dynamic state-dependent modulations and synaptic plasticity that are central to cerebellar learning. Complementary data–such as from electrophysiology or calcium imaging–would be required to verify whether structural similarity translates into shared function or temporal synchronization.

Finally, although the matching analysis effectively uncovers internal symmetry and modularity, it is primarily descriptive. Future models should integrate these similarity measures into generative frameworks or predictive models of cerebellar computation. In doing so, it may become possible to link matching profiles directly to behavioral phenotypes or disease-related disruptions in cerebellar circuitry.

In sum, the connectivity matching analysis elucidates the fine-scale architecture of the cerebellar connectome and supports a model in which the cerebellum combines structural repetition with functional specialization. This balance likely underlies the cerebellum’s capacity for parallel processing, adaptation, and robust control across cognitive, motor, and autonomic domains.

### Communicability and Information Flow in the Cerebellar Connectome

The communicability analysis of the cerebellar connectome reveals important aspects of how structural pathways enable potential information flow within and beyond the cerebellum. Communicability, defined through matrix exponential formulations of the adjacency matrix, reflects the number and efficiency of possible walks between node pairs–thereby indicating how easily regions can interact through direct and indirect paths [100, 109, 111, 132, 133].

Within the cerebellum, high communicability values were observed between deep nuclei (e.g., dentate, fastigial) and neighboring cortical zones, particularly in lobules VI–VIII. These findings align with known cortico-nuclear loops and highlight the cerebellum’s efficiency in implementing closed-loop sensorimotor and cognitive processing. Additionally, intra-lobular and bilateral cortical regions displayed strong communicability, supporting the presence of tightly integrated modular substructures and symmetrical coordination across hemispheres.

Despite these internal peaks, cerebellar regions generally exhibited lower overall communicability (both input and output) compared to non-cerebellar regions. This distinction points to a more functionally specialized and compartmentalized role for the cerebellum–suggesting that, rather than serving as a global broadcast hub, it participates in more context-specific, recursive communication loops.

The cerebellum’s reduced communicability variance, compared to the broader cortex, further implies a streamlined architecture optimized for specific timing and control functions. However, the presence of non-negligible communicability between cerebellar nuclei and distant cortical regions, particularly through the dentate-thalamic pathways, suggests that the cerebellum also supports long-range integration relevant to cognition and affect.

From a critical perspective, while communicability captures important structural tendencies, it may overestimate functional influence by including all potential walks without considering synaptic efficacy or dynamic modulation. Moreover, the high dimensionality of communicability matrices can obscure local effects, making interpretation dependent on complementary clustering or thresholding strategies.

In conclusion, the cerebellum’s communicability profile emphasizes a balance between local integration and selective global communication. This structural design may reflect an evolutionary optimization for modular processing, supporting the cerebellum’s known contributions to both motor execution and higher-order functions through tightly regulated, looped information flow.

### Topological Cohesion and Modularity in the Cerebellar Connectome

The analysis based on the generalized topological overlap matrix (GTOM) highlights key principles of cerebellar network architecture beyond immediate connectivity. GTOM quantifies higher-order topological similarity, capturing shared neighbors across multiple steps in the network. In the cerebellum, elevated average GTOM values relative to the whole brain suggest strong internal clustering and structural coherence, likely supporting the cerebellum’s well-known modular and bilaterally organized architecture [100, 109, 127, 128, 134].

Hierarchical clustering based on GTOM similarity revealed six cerebellar submodules, consistent with anatomical and functional subdivisions. Although the modularity score (*Q = 0.097*) was modest, this likely reflects overlapping or gradient-like transitions between modules rather than a lack of organization. The dendrogram and t-SNE embedding confirmed that cerebellar regions form a relatively distinct and compact topological subnetwork, further supporting their unique embedding in the brain’s connectome.

Compared to simpler metrics like connectivity matching, GTOM proved more robust and informative by capturing both direct and indirect network relationships. This was especially relevant for identifying integrative regions and detecting functionally cohesive groups despite potential noise or local variability in connectivity data.

However, while GTOM captures structural modularity effectively, it does not directly reflect temporal or functional dynamics. Its static nature limits inference about real-time communication or plasticity. Additionally, the modest modularity score, although interpretable in biological terms, may complicate efforts to define crisp, non-overlapping functional boundaries within the cerebellum.

Nonetheless, GTOM-based analyses offer a powerful lens for understanding mesoscale cerebellar organization. The findings support the view that cerebellar circuits are not only spatially segregated but also topologically cohesive, with redundancy and shared embedding likely contributing to resilience and parallel computation. These properties make GTOM a valuable tool for extending connectomic analyses to dynamic modeling and disease studies where topological robustness is relevant.

### Network Fragility and Functional Specialization Revealed by Vulnerability Analysis

The vulnerability analysis provides compelling evidence for the structural fragility and functional specialization of the cerebellar connectome. By systematically removing edges ranked by significance, we observed that the empirical network’s clustering coefficient declines sharply and irregularly when high-significance edges are removed (green curve), while low-significance edge removal has a substantially weaker effect (blue curve). In contrast, surrogate rewiring networks exhibited smooth and gradual clustering decay under both conditions, indicating a more redundant and topologically uniform structure [98, 109, 135–137].

These findings suggest that the cerebellar network architecture is non-randomly organized and critically dependent on a small subset of structurally essential edges. The abrupt decline and irregular oscillations in clustering reflect a vulnerability to targeted disruptions and imply the existence of edge-level "connectivity bottlenecks" that sustain local modular cohesion. Importantly, such a pattern was absent in surrogate networks, reinforcing the interpretation that high-significance edges play a topologically privileged role in preserving functional integration.

From a neurobiological standpoint, this asymmetric vulnerability aligns with the cerebellum’s role in high-precision coordination tasks, where tightly organized and efficiently routed processing pathways are crucial. The results point toward an evolutionarily refined architecture optimized for reliability and efficiency, but potentially susceptible to targeted damage or pathological disconnection.

A limitation of this approach is its reliance on edge significance measures that may conflate anatomical reliability with functional importance. Moreover, vulnerability here is operationalized via the clustering coefficient–a measure that emphasizes local structure but may not fully capture global network integrity or compensatory routing. Future extensions could include alternative robustness metrics such as path length, efficiency, or communicability decay to enrich the understanding of cerebellar resilience.

Overall, the vulnerability profile of the cerebellar connectome underscores its delicate balance between efficiency and fragility, highlighting the disproportionately large structural and functional impact of a small fraction of highly significant edges.

## Conclusion

This study presents a comprehensive systems-level analysis of the cerebellar connectome, leveraging high-resolution tract-tracing data and graph-theoretical frameworks to explore its structural organization, modularity, and vulnerability. By integrating mesoscale network modeling with motif, matching, communicability, and topological overlap analyses, we reveal that the cerebellar network is characterized by strong internal modularity, lateralized symmetry, and a non-random architecture optimized for both efficiency and specialization.

Key findings include: (i) the identification of distinct functional submodules within the cerebellar network through GTOM and modularity analysis; (ii) the detection of recurrent and overrepresented triadic motifs that likely support feedback and error-correction mechanisms; (iii) the discovery of structurally central regions in the brainstem and cerebellar nuclei through local connectome metrics; and (iv) a pronounced vulnerability to the removal of high-significance edges, indicating a reliance on a small subset of critical connections for maintaining local clustering and topological integrity.

Importantly, the results demonstrate that cerebellar regions–while structurally embedded in a larger brain network–form a topologically cohesive and functionally differentiated subnetwork. The balance between redundancy, modularity, and specialized motifs suggests a refined organizational logic tuned for parallel computation, precision timing, and integrative control.

Limitations include the reliance on static structural data, the absence of temporal dynamics, and the abstraction from cellular-level specificity. Nevertheless, the modeling approach offers a reproducible and biologically plausible framework for future simulation, multimodal integration, and comparative connectomics. Overall, the findings support a paradigm in which the cerebellum contributes not only to sensorimotor control, but also to higher-order functions via structurally embedded, topologically optimized networks.

## Data Availability

https://figshare.com/articles/dataset/Cerebellar_connectome/29528057, 10.6084/m9.figshare.29528057

## Appendix A List of Abbreviations

**Table:**

Abbr.	Definition	Abbr.	Definition
10	Dorsal motor nucleus of vagus	12M	Hypoglossal motor nucleus
12periC	Perihypoglossal complex	5SSC	Trigeminal subnucleus caudalis
A1	A1 noradrenergic cells	A35	Perirhinal cortex
A5	A5 noradrenaline cells	AHA	Anterior hypothalamic area
AID	Agranular insular cortex dorsal part	AINMC	Caudal part of the anterior interposed nucleus medial part
AINR	Anterior interposed nucleus rostral part	AIV	Agranular insular cortex ventral part
AM	Anteromedial thalamic nucleus	AnV	Anteroventral preoptic nucleus
AP	Area postrema	ArcM	Arcuate nucleus medial part
B	Basal nucleus Meynert	Bask	basket neurons
BL	Basolateral amygdaloid nucleus	BPNdL	Pontine nuclei dorsolateral part
BPNL	Pontine nuclei lateral part	BPNm	Pontine nucleus medial part
BPNvM	Medial ventral pontine region	Brush	unipolar brush neurons
BST	Bed nucleus of the stria terminalis	CA1	Field CA1 of hippocampus
CA2	Field CA2 of hippocampus	CA3	Field CA3 of hippocampus
CAIN	Central part of anterior interposed nucleus	CeL	Central amygdaloid nucleus lateral division
CeM	Central amygdaloid nucleus medial division	CERL10aC1	Cerebellar lobule 10a zone C1
CERL10aC2	Cerebellar lobule 10a zone C2	CERL10aC3	Cerebellar lobule 10a zone C3
CERL10aD1	Cerebellar lobule 10a zone D1	CERL10aD2	Cerebellar lobule 10a zone D2
CERL10bC1	Cerebellar lobule 10b zone C1	CERL10bC2	Cerebellar lobule 10b zone C2
CERL10bC3	Cerebellar lobule 10b zone C3	CERL10bD1	Cerebellar lobule 10b zone D1
CERL10bD2	Cerebellar lobule 10b zone D2	CERL1C1	Cerebellar lobule 1 zone C1
CERL1C2	Cerebellar lobule 1 zone C2	CERL1C3	Cerebellar lobule 1 zone C3
CERL1D2	Cerebellar lobule 1 zone D2	CERL2aC1	Cerebellar lobule 2a zone C1
CERL2aC2	Cerebellar lobule 2a zone C2	CERL2aC3	Cerebellar lobule 2a zone C3
CERL2aD2	Cerebellar lobule 2a zone D2	CERL2bC1	Cerebellar lobule 2b zone C1
CERL2bC2	Cerebellar lobule 2b zone C2	CERL2bC3	Cerebellar lobule 2b zone C3
CERL2bD2	Cerebellar lobule 2b zone D2	CERL3aC1	Cerebellar lobule 3a zone C1
CERL3aC2	Cerebellar lobule 3a zone C2	CERL3aC3	Cerebellar lobule 3a zone C3
CERL3aD2	Cerebellar lobule 3a zone D2	CERL3bC1	Cerebellar lobule 3b zone C1
CERL3bC2	Cerebellar lobule 3b zone C2	CERL3bC3	Cerebellar lobule 3b zone C3
CERL3bD2	Cerebellar lobule 3b zone D2	CERL4C1	Cerebellar lobule 4 zone C1
CERL4C2	Cerebellar lobule 4 zone C2	CERL4C3	Cerebellar lobule 4 zone C3
CERL4D2	Cerebellar lobule 4 zone D2	CERL5C1	Cerebellar lobule 5 zone C1
CERL5C2	Cerebellar lobule 5 zone C2	CERL5C3	Cerebellar lobule 5 zone C3
CERL5D2	Cerebellar lobule 5 zone D2	CERL6aB	Cerebellar lobule 6a zone B
CERL6aC1	Cerebellar lobule 6a zone C1	CERL6aC2	Cerebellar lobule 6a zone C2
CERL6aC3	Cerebellar lobule 6a zone C3	CERL6aD0	Cerebellar lobule 6a zone D0
CERL6aD2	Cerebellar lobule 6a zone D2	CERL6bC1	Cerebellar lobule 6b zone C1
CERL6bC2	Cerebellar lobule 6b zone C2	CERL6bC3	Cerebellar lobule 6b zone C3
CERL6bD2	Cerebellar lobule 6b zone D2	CERL6cC1	Cerebellar lobule 6c zone C1
CERL6cC2	Cerebellar lobule 6c zone C2	CERL6cC3	Cerebellar lobule 6c zone C3
CERL6cD0	Cerebellar lobule 6c zone D0	CERL6cD1	Cerebellar lobule 6c zone D1
CERL6dC1	Cerebellar lobule 6d zone C1	CERL6dC2	Cerebellar lobule 6d zone C2
CERL6dC3	Cerebellar lobule 6d zone C3	CERL6dD2	Cerebellar lobule 6d zone D2
CERL7aC2	Cerebellar lobule 7a zone C2	CERL7aC3	Cerebellar lobule 7a zone C3
CERL7aD1	Cerebellar lobule 7a zone D1	CERL7aD2	Cerebellar lobule 7a zone D2
CERL7bC2	Cerebellar lobule 7b zone C2	CERL7bC3	Cerebellar lobule 7b zone C3
CERL7bD0	Cerebellar lobule 7b zone D0	CERL7bD1	Cerebellar lobule 7b zone D1
CERL7bD2	Cerebellar lobule 7b zone D2	CERL7zD	Zone D of cerebellar lobule 7
CERL8C1	Cerebellar lobule 8 zone C1	CERL8C2	Cerebellar lobule 8 zone C2
CERL8C3	Cerebellar lobule 8 zone C3	CERL8D1	Cerebellar lobule 8 zone D1
CERL8D2	Cerebellar lobule 8 zone D2	CERL9aC2	Cerebellar lobule 9a zone C2
CERL9aC3	Cerebellar lobule 9a zone C3	CERL9aD1	Cerebellar lobule 9a zone D1
CERL9aD2	Cerebellar lobule 9a zone D2	CERL9bC2	Cerebellar lobule 9b zone C2
CERL9bC3	Cerebellar lobule 9b zone C3	CERL9bD1	Cerebellar lobule 9b zone D1
CERL9bD2	Cerebellar lobule 9b zone D2	CERL9cC2	Cerebellar lobule 9c zone C2
CERL9cC3	Cerebellar lobule 9c zone C3	CERL9cD1	Cerebellar lobule 9c zone D1
CERL9cD2	Cerebellar lobule 9c zone D2	CERLD	Dorsal part of lateral cerebellar nucleus
CERLdl	Lateral cerebellar nucleus dorsolateral magnocellular portion	CERLrtt	Lateral cerebellar nucleus rostral two-thirds
CERpinR	Posterior interposed nucleus rostral part	CeZA	A-zone of the cerebellar cortex
CeZA1	A1-zone of the cerebellar cortex	CeZC3	C3-zone of the cerebellar cortex
Cg	Cingulate cortex	CGA	Central gray alpha part
CGMint	Central gray mesencephalic part intercollicular level	CGP	Central gray pons part
CGVD	Central gray lateral ventral part	CL	Centrolateral thalamic nucleus
CLi	Caudal linear nucleus of the raphe	CLILN	Centrolateral intralaminar nucleus
CM	Central medial thalamic nucleus	CnF	Cuneiforme nucleus
Com	Commissural nucleus of the inferior colliculus	CopC1mHp	Copula of the pyramis C1 zone medial hindpaw zone
CopM	Medial part of the copula of the pyramis	CoSeg	Coccygeal segments
CPudl	Dorsolateral striatum	Crus2m	Ansiform lobule crus 2 medial part
CS	Superior central raphe nucleus	CS1	Cervical segment 1
CS2	Cervical segment 2	CS3	Cervical segment 3
CS4	Cervical segment 4	CS5	Cervical segment 5
CS6	Cervical segment 6	CS7	Cervical segment 7
CS8	Cervical segment 8	cSC	Superior colliculus caudal part
CuC	Cuneate nucleus caudal part	CuLa	Cuneate nucleus lateral part
CuM	Middle part of cuneate nucleus	CuMED	Cuneate nucleus medial part
CuVL	Cuneate nucleus ventrolateral part	CVLMl	Caudal ventrolateral medulla lateral part
DA	Dorsal hypothalamic area	DGL	Dentate gyrus layers
DHR	Dorsal region	Dk	Nucleus of Darkschewitsch
DLHCERL	Dorsolateral hump of the lateral cerebellar nucleus	DLPr	Dorsolateral protuberance
DMDM	Dorsomedial hypothalamic nucleus dorsomedial part	DMH	Dorsomedial hypothalamic nucleus diffuse part
DMHp	Dorsomedial nucleus posterior part	DMHyA	Dorsomedial hypothalamic area
DMPn	Dorsomedial pontine nucleus	DNC	deep cerebellar nuclei
DoLaP	Dorsolateral pons	DPCRt	Dorsal parvocellular reticular formation
DPGi	Dorsal paragigantocellular nucleus	DpMei	Deep mesencephalic nucleus intermediate
DpMeL	Deep mesencephalic nucleus lateral part	DpMeM	Deep mesencephalic nucleus medial part
DPPn	Dorsal peduncular pontine nucleus	DRF	Dorsal reticular formation
DRNM	Dorsal raphe nucleus medial part	DRPo	Dorsal raphe nucleus pontine part
DTg	Dorsal tegmental nucleus	Ent	Entorhinal cortex
FFH1	Forel field H1	FMCx	Forelimb motor cortex
FPl	Frontal polar cortex lateral part	GI	Granular insular cortex
Gidp	Gigantocellular reticular nucleus dorsal part	GirL	Gigantocellular reticular nucleus lateral part
GiV	Gigantocellular reticular nucleus ventral part	Golgi	Golgi neurons
Gr	Gracile nucleus principal part	GraCb	granule neurons
HMCx	Hindlimb motor cortex	ICNL	Lateral interposed cerebellar nucleus
ICNM	Interposed cerebellar nuclei medial part	IF	Interfascicular nucleus
IL	Infralimbic cortex	ILNA	Intralaminar nuclei anterior part
IM	Intercalated masses	In	Intercalated nucleus of the medulla
Inf	Infracerebellar nucleus	IntDL	Interposed cerebellar nucleus dorsolateral hump
InWh	Intermediate white layer of the superior colliculus	IODC	Inferior olive dorsal nucleus caudal part
IODcl	Inferior olive dorsal nucleus caudolateral part	IOdo	Inferior olive medial nucleus dorsal part
IODRL	Lateral part of the rostral inferior olive dorsal nucleus	IODvLM	Medial portion of the inferior olive dorsal nucleus ventral leaf
IOL	Inferior olive lateral part	IOMch	Caudal half of the inferior olive medial nucleus
IOMcLa	Lateral part of the inferior olive medial nucleus caudal part	IOMm	Inferior olive medial nucleus medial part
IOMVe	Ventral portion of the inferior olive medial nucleus	IOPrMeh	Medial half of the inferior olive principal nucleus ventral lamella
IOPrvlLh	Lateral half of the ventral lamella of the inferior olive principal nucleus	IP	Interpeduncular nucleus
IRt	Intermediate reticular nucleus	KF	Koelliker Fuse nucleus
LAH	Lateroanterior hypothalamic nucleus	LCD	Locus coeruleus dorsal part
LCRo	Locus coeruleus rostral part	LCv	Locus coeruleus ventral part
LDTg	Laterodorsal tegmental nucleus	LGP	Lateral globus pallidus
LHb	Lateral habenular nucleus	LHLZM	Lateral hypothalamic area [Lateral zone mammillary level]
LHN	Lateral hypothalamic nucleus	LM	Lateral mammillary nucleus
LP	Lateral posterior thalamic nucleus	LPAG	Lateral periaqueductal gray
LPBL	Lateral parabrachial nucleus lateral part	LPGi	Lateral paragigantocellular nucleus
LPn	Lateral pontine nucleus	LPN	Lateral preoptic nucleus
LPO	Lateral preoptic area	LRtV	Lateral reticular nucleus ventral part
LSD	Lateral septal nucleus dorsal part	LSeg	Lumbar segments
LSI	Lateral septal nucleus intermediate part	LTeN	Lateral terminal nucleus of the accessory optic tract
LTN	Lateral tegmental nucleus	Lug	Lugaro neurons
LVeCa	Lateral vestibular nucleus caudal part	LVERM	Lateral vermis
LVeRV	Lateral vestibular nucleus rostroventral part	LVeV	Lateral vestibular nucleus ventral part
MA3	Medial accessory oculomotor nucleus	MdH	Medial division of the hypothalamus
MDL	Mediodorsal thalamic nucleus lateral part	MDTV	Mediodorsal thalamic nucleus ventral part
MdV	Medullary reticular nucleus ventral part	MdVLR	Medullary reticular nucleus ventrolateral part rostral part
Me5cp	Mesencephalic trigeminal nucleus caudal pole	MEA	Midbrain extrapyramidal area
MeAD	Medial amygdaloid nucleus anterodorsal part	Medc	Medial cerebellar nucleus caudal part
MedCMV	Ventral part of the caudomedial subdivision of the medial cerebellar nucleus	Medim	Medial cerebellar nucleus intermediate part
MedL	Medial cerebellar nucleus lateral part	Medmh	Medial cerebellar nucleus medial half
MedR	Medial cerebellar nucleus rostral part	MedV	Medial cerebellar nucleus ventral part
MeObL	Medulla oblongata lateral part	MePD	Medial amygdaloid nucleus posterodorsal part
MePG	Medial pontine gray	MES	spinal cord
MGP	Medial globus pallidus	MM	Medial mammillary nucleus medial part
MMdD	Medial medullary reticular nucleus	Mo5	Motor trigeminal nucleus
MPA	Medial preoptic area	MpLRt	Midportion of the lateral reticular nucleus
MPO	Medial preoptic nucleus	MPoN	Medial pontine nucleus
MPYR	Medial part of the pyramis	mSC	Superior colliculus medial part
MSim	Medial portion of the simple lobule	MT	Medial terminal nucleus of the accessory optic tract
MVeCPC	Medial vestibular nucleus caudal parvicellular part	MVeD	Medial vestibular nucleus dorsal part
MVeM	Medial vestibular nucleus medial part	MVeMC	Medial vestibular nucleus magnocellular part
MVePCrcVM	Ventromedial area of the rostrocaudal extent of the parvicellular medial vestibular nucleus	MVeVL	Medial vestibular nucleus ventrolateral part
Name	Name	Nic	Nucleus intercalatus
NLL	Nuclei of the lateral lemniscus	NRMC	Magnocellular reticular nucleus
NRTPc	Pontine reticular nucleus central part	OrC	Orbital cortex
OSA	Olfacto septal area	OT	Nucleus of the optic tract
P	pons	Pa5	Paratrigeminal nucleus
PaDC	Paraventricular hypothalamic nucleus dorsal cap [Periventricular zone anterior region]	PAGdlc	Dorsolateral periaqueductal gray caudal part
PAGdlr	Dorsolateral periaqueductal gray rostral part	PAGvlc	Ventrolateral periaqueductal gray caudal part
PAGvlr	Ventrolateral periaqueductal gray rostral part	PaR	Pararubral nucleus
PBD	Parabrachial nucleus dorsal part	PBnMe	Parabrachial nucleus medial
PBveLa	Parabrachial nucleus ventrolateral part	PBVL	Parabrachial nucleus ventral part lateral portion
PC	Paracentral thalamic nucleus	PcaVL	Caudal pons ventrolateral group
PCom	Nucleus of the posterior commissure	PCRtl	Parvicellular reticular nucleus lateral part
Pe	Periventricular hypothalamic nucleus	PeF	Perifornical nucleus
PFa	Parafascicular nucleus	PFCM	Parafascicular centre-median complex
PFlR	Rostral paraflocculus	PFlvM	Medial ventral paraflocculus
PFPR	Parafascicular thalamic nucleus prerubral part	PFx	Lateral hypothalamic area perifornical part
PH	Posterior hypothalamic nucleus	PHA	Posterior hypothalamic area
Pir	Piriform cortex	PkPC	Purkinje neurons
PLCo	Posterolateral cortical nucleus	PLH	Peduncular part of lateral hypothalamus
PMAN	Premammillary nucleus	PMn	Paramedian reticular nucleus
PN	Paranigral nucleus	PnO	Pontine reticular nucleus oral part
PnR	Pontine raphe nucleus	PnV	Pontine reticular nucleus ventral part
Po	Posterior thalamic nuclear group	PoMn	Posteromedian thalamic nucleus
PPdo	Peripeduncular nucleus dorsal part	PPT	Posterior pretectal nucleus
PPTg	Pedunculopontine tegmental nucleus	PPv	Peripeduncular nucleus ventral part
PR	Prerubral field	Pr5ca	Principal sensory trigeminal nucleus caudal part
Pr5D	Principal sensory trigeminal nucleus dorsal part	Pr5DL	Principal sensory trigeminal nucleus dorsolateral part
Pr5v	Principal sensory trigeminal nucleus ventral part	pRNc	Parvicellular reticular nucleus caudal portion
PSol	Parasolitary nucleus	PtA	Parietal association cortex
PVA	Paraventricular thalamic nucleus anterior part	PVG	Periventricular gray matter
RFm	Medial reticular formation	RFMED	Median reticular nucleus
RFN	Retrofacial nucleus	Rh	Rhomboid thalamic nucleus
RLi	Rostral linear nucleus of the raphe	Rmed	Red nucleus medial part
RMeObvm	Rostral part of the ventromedial medulla oblongata	RMgc	Raphe magnus nucleus caudal part
RMgR	Raphe magnus nucleus rostral part	RMRf	Rostral mesencephalic reticular formation
RMTg	Rostromedial tegmental nucleus	Ro	Nucleus of Roller
RObc	Raphe obscurus nucleus caudal part	RPa	Raphe pallidus nucleus
RRFL	A8 dopamine cells retrorubral group lateral part	RRFm	A8 dopamine cells retrorubral group medial part
RTN	Retrotrapezoid nucleus	Rtr	Reticular thalamic nucleus rostral part
RtTgD	Dorsal part of the reticulotegmental nucleus of the pons	RV	Red nucleus ventral part
RVb	Nucleus reticular ventralis pars beta	RVRG	Rostral ventral respiratory group
S1BF	Primary somatosensory cortex barrel field	S1FP	Primary somatosensory cortex forelimb region forepaw
S1HL	Primary somatosensory cortex hindlimb region	S2FP	Forepaw area of secondary somatosensory cortex
S2OFWP	Secondary somatosensory cortex orofacial region whisker representation	SGe	Supragenual nucleus
SHy	Septohypothalamic nucleus	SID	Substantia innominata dorsal part
SNCM	Substantia nigra compact part medial tier	SNL	Substantia nigra lateral part
SNR	Substantia nigra reticular part	SolC	Nucleus of the solitary tract commissural part
SolIM	Nucleus of the solitary tract intermediate part	SolM	Nucleus of the solitary tract medial part
SolRL	Nucleus of the solitary tract rostrolateral part	Sp5CC	Caudal part of the spinal trigeminal nucleus caudal part
Sp5Ib	Spinal trigeminal nucleus interpolar part border region	Sp5ID	Spinal trigeminal nucleus interpolar part dorsal part
Sp5IdL	Spinal trigeminal nucleus interpolar part dorsolateral	Sp5IDM	Spinal trigeminal nucleus interpolar part dorsomedial part
Sp5Ir	Spinal trigeminal nucleus interpolar part rostral	Sp5Oc	Caudal part of spinal trigeminal nucleus oral part
Sp5OD	Dorsal part of spinal trigeminal nucleus oral part	Sp5OV	Ventral part of spinal trigeminal nucleus oral part
SPF	Subparafascicular thalamic nucleus	SPTg	Subpeduncular tegmental nucleus
SpVeC	Spinal vestibular nucleus caudal part	SpVeL	Spinal vestibular nucleus lateral part
SpVeR	Spinal vestibular nucleus rostral part	SRD	Subnucleus reticularis dorsalis
SSeg	Sacral segments	Ste	stellate neurons
STh	Subthalamic nucleus	SubCD	Subcoeruleus nucleus dorsal part
T1	Thoracal segment 1	T2	Thoracal segment 2
T3	Thoracal segment 3	T4	Thoracal segment 4
TBMdm	Tuberomammillary nucleus dorsomedial part	TC	Tuber cinereum area
TGmi	Midbrain tegmentum	TSegLo	Lower thoracic spinal cord
TSegM	Mid thoracal segments	UVUaS	Superficial part of cerebellar lobule 9a
UVUbS	Superficial part of cerebellar lobule 9b	UVUcS	Superficial part of cerebellar lobule 9c
VBPNc	Ventral pontine nucleus caudalis	VERMM	Midline cerebellar vermis
VLGM	Ventral lateral geniculate nucleus medial part	VLGPC	Ventral lateral geniculate nucleus parvicellular part
VLLa	Ventrolateral thalamic nucleus lateral part	vlPRF	Ventrolateral pontine reticular formation
VLRF	Ventrolateral reticular formation	VLRo	Ventrolateral thalamic nucleus rostral part
VLTg	Ventrolateral tegmental area	VMctt	Ventromedial thalamic nucleus caudal two thirds
VMeO	Ventral medulla oblongata	VMH	Ventromedial hypothalamic nucleus
VML	Ventromedial thalamic nucleus lateral part	VMSe	Ventral medial septum
VMT	Ventral medial nucleus of the thalamus	Vncf	Subnucleus f
VP	Ventral pallidum	VPAG	Ventral periaqueductal gray
VPLro	Ventral posterolateral thalamic nucleus rostral part	VPMm	Medial part of ventral posteromedial thalamic nucleus
VPMpc	Ventral posteromedial thalamic nucleus parvicellular part	VPTm	Ventral posterior thalamic complex medial part
VTAdLdv	Ventral tegmental area A10 dorsolateral part dorsal periventricular part	VTAL	Ventral tegmental area A10 lateral part
VTAR	Ventral tegmental area rostral part	VTg	Ventral tegmental nucleus
VTM	Ventral tuberomammillary nucleus	VTRZ	Ventral tegmental relay zone
X	Nucleus X	XVES	X cell group of the vestibular complex
ZIC	Zona incerta caudal part	ZIm	Zona incerta medial part
ZIV	Zona incerta ventral part		

### A.0.1 Local Connectome Analysis

The present analysis involves a systematic evaluation of local connectome parameters computed across cerebellar regions, based on the cerebellar connectome dataset sorted by average rank. Each region’s position and functional importance are interpreted through a set of 51 connectome metrics. For clarity, these metrics were grouped into coherent categories according to their theoretical properties and functional implications in connectomics.

The selection of the 11 specific local connectome parameters presented in Table 2, out of a total of 51 calculated metrics, is methodologically deliberate and grounded in both theoretical and statistical reasoning. This subset captures the most essential and distinct dimensions of nodal connectivity and influence within the cerebellar connectome. The rationale for this selection is outlined in the following.

The 11 parameters presented in Table 2 were selected based on theoretical representativeness, avoidance of redundancy, statistical sufficiency, neurobiological interpretability, and practical display constraints (see appendix  8). The 5 subsets enable accurate and region-specific analysis of local connectivity while ensuring that the full complexity of the cerebellar connectome is meaningfully summarized.

The group of degree centrality and local topology metrics include DG$_\textrm{All}$, DG$_\textrm{Out}$, DG$_\textrm{In}$, CDC, Lat$_\textrm{All}$, Lat$_\textrm{Out}$, Lat$_\textrm{In}$, CluC$_2$, AvgDG$_\textrm{nb}$, VC$_\textrm{DG}$, Hub, Aut. This group encompasses measures reflecting the number and nature of a node’s connections. High values of total degree (DG$_\textrm{All}$) and directional degrees (DG$_\textrm{In}$, DG$_\textrm{Out}$) identify key connectivity hubs. Notable among these are the *Pedunculopontine tegmental nucleus* (left and right), *Parabrachial nucleus medial* (L/R), and *Pontine reticular nucleus oral part*. The *Convergent-Divergent Coefficient (CDC)* distinguishes between incoming and outgoing bias. Most high-ranking regions show CDC near 0.5, indicating balanced connectivity. The *laterality measures* (Lat$_\textrm{All}$, Lat$_\textrm{Out}$, Lat$_\textrm{In}$) show ipsilateral dominance in most nodes, consistent with cerebellar hemispheric specialization. The *Clustering coefficient (CluC* $_2$ *)* and *neighborhood degree (AvgDG* $_\textrm{nb}$ *)* point to locally clustered and integrated subnetworks. A high *Hub* and *Authority (Aut)* scores further establish these regions as topological and influential hubs.

The group of participation and modularity contains *PC* $_\textrm{All}$ , *PC* $_\textrm{Out}$ and *PC* $_\textrm{In}$ . These metrics quantify how well a region distributes its connections across different modules in the connectome. A *PC* value near 0.5 reflects balanced inter-modular interaction, neither strictly local nor globally distributed. The *parabrachial nuclei* and the *pontine nuclei* exhibit intermediate PC values, suggesting roles in cross-modular integration without strict modular confinement.

Then the parameter group of radial and spatial embedding metrics include Rad$_\textrm{Out}$, Rad$_\textrm{In}$, Cen$_\textrm{Out}$ and Cen$_\textrm{In}$. This group captures the spatial and topological embedding of nodes within the entire connectome. *Radiality* values were consistently high across central regions, indicating that these nodes are topologically close to most other nodes. *Centroid* values (more negative indicating peripheral location) help spatially locate these nodes. For instance, *Pontine nuclei* exhibit moderately negative centroid values, aligning with an intermediary role in information relay.

The parameter class of controllability and connectome role metrics contains *Knot* and *MC* (Modal controllability). This category pertains to the influence a node has over connectome dynamics. The *Knot* metric is zero for nearly all regions, indicating absence from the densely interconnected “knotty” center. *Modal Controllability (MC)* values are largely negative, especially in key brainstem regions like the *Pedunculopontine nucleus*, suggesting limited capacity for initiating state transitions–yet a potential role in stabilizing dynamics.

Global contribution and vulnerability parameters includes *AvgRank*, *Lev*, *CC* $_\textrm{Out}$ ,* CC* $_\textrm{In}$ and *Sig*. These metrics reflect the node’s contribution to overall connectome performance and robustness.*Average rank (AvgRank)* offers a global composite score. Top-ranked nodes include bilateral *Pedunculopontine* and *Pontine reticular* nuclei. *Closeness centrality (CC)* metrics highlight accessibility; higher values correlate with efficient information propagation. *Sig* quantifies the connectome impact of removing a node. High Sig scores in nodes like the *Parabrachial nucleus* and *Pontine reticular nuclei* imply they are critical for maintaining connectome cohesion and performance.

**Table 5:** Local network parameters of high ranked cerebellar connectome regions. Top 30 from a total of 862 regions of the cerebellar connectome sorted by their average ranks over all normalized 51 parameters. The table presents key local parameters characterizing node importance, connectivity, modular integration, spatial embedding, and vulnerability. *DG*: Number of input and output connections of a node, *CDC* :  Convergent divergent coefficient (in degree divided by degree), $CluC_2:$ Hierarchical cluster coefficient of level 2: Edge count between all neighbours (2nd degree) / largest possible number, *Hub* :  Hubness Hub of a node. Degree to which a node has output to authorities [138], $PC_{All}:$ Participation coefficient [139]: If PC=0, all input and output edges of this node are in one cluster. Higher values indicate that this node has more connections, $Rad_{Out}:$ Output radiality of a node. Peripheral nodes have smaller and central nodes have larger values, $Cen_{Out}:$ Output centroid value of a node., *MC* :  Modal controllability which identifies brain areas that can push a brain into a difficult-to-reach states [140], *Sig* :  Negative related change of the average closeness removing a node (in percent). A value of 10 means that average closeness decreases by 10% and so the average pathlength in the connectome increases if the node is removed. A negative value indicate that the average pathlength decreases if a node is removed, *AvgRank* :  Average rank is a composite indicator that summarizes a region’s importance across all order preserved, sign corrected and normalized connectome metrics

Region	DG$_{\text {All}}$	CDC	CluC$_2$	Hub	PC$_{\text {All}}$	Rad$_{\text {Out}}$	Cen$_{\text {Out}}$	MC	Sig	AvgRank
Pedunculopontine_tegmental_nucleus_R	287	0.477	0.01	0.839	0.547	9.684	-140	-112.75	0.188	190.42
Pontine_reticular_nucleus_oral_part_L	224	0.54	0.014	0.74	0.549	9.602	-212	-84.5	0.127	190.68
Pontine_reticular_nucleus_oral_part_R	224	0.54	0.014	0.74	0.549	9.602	-212	-84.5	0.127	190.68
Pedunculopontine_tegmental_nucleus_L	287	0.477	0.01	0.839	0.547	9.683	-141	-112.75	0.188	190.76
Nucleus_of_Darkschewitsch_R	127	0.52	0.023	0.535	0.503	9.43	-360	-50.25	0.216	191.28
Nucleus_of_Darkschewitsch_L	127	0.52	0.023	0.535	0.503	9.43	-360	-50.25	0.215	191.42
Parabrachial_nucleus_medial_L	234	0.47	0.018	0.744	0.509	9.648	-172	-92.0	0.164	192.36
Parabrachial_nucleus_medial_R	234	0.47	0.018	0.744	0.509	9.648	-172	-92.0	0.164	192.36
Lateral_paragigantocellular_nucleus_L	209	0.512	0.016	0.734	0.482	9.602	-212	-83.75	0.128	195.5
Lateral_paragigantocellular_nucleus_R	209	0.512	0.016	0.734	0.482	9.602	-212	-83.75	0.128	195.62
Laterodorsal_tegmental_nucleus_R	277	0.444	0.014	0.847	0.481	9.713	-115	-109.75	0.17	197.46
Laterodorsal_tegmental_nucleus_L	277	0.444	0.014	0.847	0.481	9.713	-115	-109.75	0.17	197.66
Bed_nucleus_of_the_stria_terminalis_R	276	0.493	0.024	0.574	0.463	9.6	-212	-108.0	0.341	198.26
Bed_nucleus_of_the_stria_terminalis_L	276	0.493	0.023	0.574	0.463	9.6	-212	-108.0	0.341	198.28
Posterior_hypothalamic_area_R	191	0.471	0.021	0.508	0.522	9.567	-241	-68.75	0.112	198.7
Posterior_hypothalamic_area_L	191	0.471	0.021	0.508	0.522	9.567	-241	-68.75	0.112	198.76
Lumbar_segments_R	329	0.523	0.012	0.992	0.467	9.847	0	-125.75	0.392	199.76
Infralimbic_cortex_L	267	0.461	0.012	0.65	0.486	9.728	-102	-102.75	0.523	199.86
Infralimbic_cortex_R	267	0.461	0.012	0.65	0.486	9.728	-102	-102.75	0.523	199.88
Lumbar_segments_L	329	0.523	0.012	0.992	0.463	9.847	0	-125.75	0.392	199.94
Thoracal_segment_3_R	228	0.566	0.015	0.84	0.398	9.684	-140	-87.5	0.125	200.98
Thoracal_segment_3_L	228	0.566	0.015	0.84	0.398	9.684	-140	-87.5	0.125	201.02
Cingulate_cortex_L	278	0.428	0.011	0.862	0.465	9.823	-21	-103.5	0.359	201.1
Cingulate_cortex_R	278	0.428	0.011	0.862	0.465	9.823	-21	-103.5	0.359	201.1
Cuneiforme_nucleus_L	187	0.508	0.022	0.64	0.528	9.574	-235	-72.25	0.201	201.32
Cuneiforme_nucleus_R	187	0.508	0.022	0.64	0.528	9.574	-235	-72.25	0.201	201.44
Thoracal_segment_2_R	222	0.554	0.015	0.84	0.39	9.684	-140	-86.0	0.106	204.58
Thoracal_segment_2_L	222	0.554	0.015	0.84	0.39	9.684	-140	-86.0	0.106	204.64
Thoracal_segment_1_L	243	0.523	0.014	0.883	0.423	9.744	-88	-91.75	0.127	204.78
Cervical_segment_1_R	293	0.522	0.012	0.936	0.454	9.774	-63	-109.25	0.425	204.82

Note: All parameters are dimensionless except Radiality, Centroid (Cen), and Modal Controllability (MC). Values represent structural connectivity characteristics within the cerebellar connectome

## Appendix B Central Point Dominance (CPD)

Central Point Dominance (CPD) is a global connectome metric that quantifies the extent to which the most central node dominates the connectome in terms of betweenness centrality. CPD can attain extremely large values in connectomes where one node holds a disproportionately high share of global betweenness centrality, while the remaining nodes contribute minimally. This typically occurs in highly centralized connectomes, where shortest paths between most node pairs pass through a dominant hub. In contrast, connectomes with more homogeneous structure–such as Erdõs–Rényi graphs–distribute centrality more evenly across nodes, resulting in significantly lower CPD values. Therefore, the emergence of extremely high CPD values signals strong topological centralization and an uneven distribution of communication roles within the connectome. While this configuration can support efficient information routing, it also suggests increased vulnerability to targeted disruptions of hub nodes. The CPD metric is computed as the average difference between the maximum betweenness centrality and the betweenness centrality $C_B^{\text {max}} - C_B(i)$ of each node in the connectome. When the majority of nodes have near-zero betweenness centrality and one or a few nodes possess exceedingly high centrality, these differences become substantial. Since the formula involves summing these differences over all nodes, even small contributions accumulate rapidly in large connectomes, causing the CPD value to grow dramatically.

It is formally defined as:

$$ \text {CPD} = \frac{\sum _{i=1}^N \left( C_B^{\text {max}} - C_B(i)\right) }{N - 1} $$

where $C_B(i)$ is the betweenness centrality of node * i *, $C_B^{\text {max}}$ is the maximum betweenness centrality in the connectome, and * N * is the total number of nodes. Betweenness centrality itself measures how often a node lies on the shortest paths between all pairs of nodes, indicating its influence over the flow of information within the connectome.

A high CPD value indicates that a single node (or a small subset of nodes) controls a disproportionately large portion of information flow, effectively acting as a hub through which many shortest paths pass. Conversely, low CPD values suggest a more distributed or egalitarian structure, where no single node dominates the communication pathways.

The usefulness of CPD lies in its ability to capture hub dominance and topological centralization. It serves as a diagnostic tool to differentiate between network classes: scale-free and hierarchical models typically yield high CPD, whereas random graphs such as Erdõs–Rényi networks produce much lower values due to their uniform degree and lack of global hubs. In the context of brain connectomes, a high CPD value may reflect vulnerability to targeted attacks on hub regions, but also suggest efficient global integration and control mechanisms mediated by a few central nodes. An appendix contains supplementary information that is not an essential part of the text itself but which may be helpful in providing a more comprehensive understanding of the research problem or it is information that is too cumbersome to be included in the body of the paper.

## Appendix C Power-Law Approximation

To assess whether the empirical cerebellar connectome and various surrogate networks exhibit scale-free characteristics, we analyzed their degree distributions using a power-law approximation of the form:

$$ P(k) = \alpha \cdot k^{-\gamma } $$

Here, * P(k) * denotes the relative frequency of nodes with degree * k *, i.e., the probability that a randomly selected node in the network has exactly * k * connections. The constant * α * is a normalization factor ensuring the distribution sums to one, while * γ * is the power-law exponent that governs the decay rate of the distribution. The approximation error * Δ * is defined as the mean deviation between the empirical distribution and the fitted power-law curve, scaled by a factor of 100 for interpretability.

This analysis serves to identify whether the connectome follows a scale-free organization, a common property in many biological and technological networks. Scale-free networks are characterized by the presence of a few highly connected hub nodes and many nodes with few connections, leading to heavy-tailed degree distributions. Such topologies are associated with high resilience to random failures, efficient communication, and the emergence of hierarchical organization.

The power-law exponent * γ * controls the steepness of the decay in * P(k) *. Smaller values of * γ * (e.g., * 1< γ < 2 *) imply a higher prevalence of hubs, indicating a stronger scale-free structure. Higher * γ * values (e.g., * > 3 *) suggest a more homogeneous degree distribution, reducing hub dominance. The normalization constant * α * adjusts the vertical scaling of the distribution. While essential for curve fitting, it does not carry intrinsic structural meaning and depends primarily on the size and density of the connectome. The value * Δ * quantifies the approximation error between the observed data and the ideal power-law distribution. Lower * Δ * values indicate a better fit and thus a stronger scale-free property. High * Δ * suggests the connectome’s degree distribution deviates substantially from power-law behavior, pointing to alternate structural constraints. The degree * k * is the independent variable in the distribution. Examining how * P(k) * behaves across * k * reveals the presence (or absence) of hubs and the tail behavior of the connectome. Connectomes with small * Δ * and moderate-to-low * γ * values are more consistent with scale-free organization. Investigating these parameters across empirical and synthetic networks allows us to determine which generative models most accurately capture the hub structure and heavy-tailed nature observed in real biological systems.

## Appendix D Local Connectome Parameters and their Classification

Each parameter in the selected set represents a unique functional or structural property of a network node. Together, they provide a comprehensive yet non-redundant characterization of local connectivity. The selected parameters each quantify a distinct and functionally relevant aspect of nodal connectivity within the cerebellar connectome. Specifically, $DG_{\text {All}}$ quantifies the total number of input and output connections, thus reflecting the overall degree and communication load of a node. *CDC* captures the balance between convergent and divergent connectivity patterns by comparing in-degree to total degree, providing insight into the predominant direction of information flow.

$CluC_2$ measures local clustering at a higher hierarchical level, accounting for the connections among second-degree neighbors and thereby offering a refined view of connectome segregation. *Hub* estimates the node’s importance as a broadcaster, consistent with classical definitions of structural hubness based on connections to authoritative nodes.

$PC_{\text {All}}$ reflects the degree to which a node participates across different connectome modules; high values of this participation coefficient indicate that the node maintains intermodular connections, promoting global integration. $Rad_{\text {Out}}$ captures how centrally positioned a node is in topological space, with larger values indicating better overall access to other nodes in the connectome. Complementing this, $Cen_{\text {Out}}$ is a spatial centrality measure derived from output connectivity, reflecting a node’s embedding within the geometrical structure of the connectome.

*MC*, or modal controllability, quantifies a node’s theoretical capacity to guide the connectome into difficult-to-reach dynamic states, thus linking structure to potential functional transitions. *Sig* measures the topological vulnerability of the connectome to node deletion, specifically calculating the percentage change in average closeness centrality if the node were removed. Finally, *AvgRank* is a composite metric derived from all 51 normalized and polarity-aligned parameters; it summarizes each node’s global importance by averaging its relative rankings across the full parameter space.

These parameters span key connectome domains including integration, segregation, modularity, spatial embedding, dynamic control, and robustness, thus ensuring a well-rounded depiction of region-specific connectivity.

Many of the remaining 40 parameters are subcomponents or directional variants (e.g., in-degree, out-degree, clustering$_\text {in}$, clustering$_\text {out}$) of the included metrics. Their inclusion would introduce multicollinearity and inflate dimensionality without adding conceptual clarity. The chosen parameters avoid such redundancy by representing only one component per metric family (e.g., total degree rather than both in- and out-degree), thereby ensuring clearer interpretation and statistical independence.

Preliminary principal component analysis (PCA) and exploratory factor analysis (EFA) indicate that the first 10–12 dimensions explain the majority of the variance across nodes in the full parameter set. The selected parameters approximately correspond to these principal dimensions, ensuring that regional differentiation is preserved while reducing complexity. This supports the notion that the chosen subset is statistically sufficient for accurate node-level characterization.

The selected parameters are widely recognized in connectomics literature and have direct neurobiological interpretations. For instance, *MC* is linked to brain dynamics and cognitive control [140], $PC_{\text {All}}$ is grounded in modular integration theory [139], and *Hub* is central to structural core-periphery models. The inclusion of spatial ($Cen_{\text {Out}}$), topological ($Rad_{\text {Out}}$), and resilience (*Sig*) metrics ensures that the anatomical and functional properties of nodes are interpreted in a biologically meaningful way.

## Appendix E Motif or Subgraph Analysis

Directed 3-node motifs represent all possible ways in which three nodes can be connected with directed edges, forming a set of 13 unique triadic configurations. These motifs serve as fundamental building blocks of complex connectomes and are particularly meaningful in the context of brain architecture, where they may correspond to basic computational microcircuits. Examples include simple linear chains (e.g., A *→ * B *→ * C), closed loops (e.g., A *→ * B *→ * C *→ * A), and feedforward or feedback structures (e.g., A *→ * B, A *→ * C, B *→ * C). The functional diversity of these motifs is substantial; depending on their structure, they may support mechanisms such as information gating, signal amplification, memory retention, feedback inhibition, or oscillatory control [141].

Unlike pairwise statistics such as node degree or reciprocity, which provide information about individual or dyadic connectivity, 3-node motif analysis captures the higher-order organization of local circuits. This allows the identification of specific wiring patterns that are not apparent from traditional network metrics. For example, even when two networks share identical degree distributions, their triadic motif composition may differ substantially, reflecting different topological and functional principles. Analyzing motif distributions therefore enables researchers to detect subtle and functionally relevant structural differences between brain connectomes [142].

To determine whether observed motifs are the result of structured organization or merely random chance, it is essential to compare the empirical motif distribution to that of a suitable null model. A commonly used null model is the rewired surrogate network, which preserves key global properties of the original connectome–such as the in- and out-degree of each node–while randomizing the specific connections between nodes. This comparison allows for the quantification of motif enrichment or suppression. Motifs that occur significantly more often in the empirical connectome than in the surrogate are considered overrepresented and are interpreted as statistically meaningful and potentially functional substructures. Conversely, underrepresented motifs may indicate structural constraints or selective avoidance [143].

The biological significance of motif enrichment becomes apparent when motifs are linked to known functional mechanisms. For instance, overrepresentation of the feedforward loop motif (A *→ * B, A *→ * C, B *→ * C) may suggest an evolutionary preference for circuits that enable layered information processing and temporal integration. Similarly, enrichment of feedback motifs may relate to control loops and stability mechanisms in dynamic brain function [144].

Moreover, the distribution of 3-node motifs can provide sensitive markers for developmental and pathological changes. During brain maturation, shifts in motif composition may reflect synaptic pruning, increased modularity, or the emergence of hierarchical structure. In clinical settings, altered motif profiles have been observed in neurological and psychiatric conditions such as epilepsy, schizophrenia, and autism, making motif analysis a promising tool for characterizing connectome reorganization and dysfunction [143].

In summary, the analysis of directed 3-node motifs, particularly in comparison with rewired surrogate models, is essential for identifying higher-order architectural features that govern local processing and global dynamics in brain connectomes. This method distinguishes biologically meaningful patterns from statistical noise and offers deep insight into the principles underlying the organization, function, and development of the connectome.

## Appendix F Communicabilty Analysis

In connectomics, communicability matrices provide a quantitative framework to assess the ease with which information or signals can propagate through a connectome [145–148]. Unlike direct connectivity matrices, which represent the existence or strength of direct links between brain regions, communicability captures both direct and indirect paths by accounting for all possible walks between node pairs. Mathematically, communicability is often derived from the matrix exponential of the adjacency or Laplacian matrix, allowing the influence of longer, indirect routes to be weighted exponentially less than shorter, direct ones. This approach is particularly useful in brain connectomes, where neural communication is not restricted to monosynaptic connections but may involve complex multi-step interactions. In the context of brain architecture, high communicability between two regions implies efficient potential information flow, even if a direct connection is weak or absent. Communicability analysis has been applied to identify central hubs, functionally coherent subnetworks, and vulnerable regions within the human connectome, offering insights into the integrative properties of brain organization beyond traditional connectivity measures.

A concise formal definition of the communicability approach or matrix is given below. Let *G = (V, E)* be an undirected, unweighted graph with *n* nodes, and let $A \in \mathbb {R}^{n \times n}$ denote its adjacency matrix, where $A_{ij} = 1$ if there is an edge between nodes *i* and *j*, and $A_{ij} = 0$ otherwise. The *communicability matrix* $C \in \mathbb {R}^{n \times n}$ is defined as the matrix exponential of *A*:

$$ C = e^A = \sum _{k=0}^{\infty } \frac{A^k}{k!} $$

Each entry $C_{ij}$ of the communicability matrix quantifies the total communicability between nodes *i* and *j*, incorporating all possible walks of any length between them. The contribution of longer walks is down-weighted by a factor of 1/*k*!, ensuring that shorter paths contribute more significantly to the overall communicability. This formulation allows the matrix *C* to capture not only direct but also indirect interactions between all pairs of nodes in the connectome.

## Appendix G Generalized Topology Matrix (GTOM)

The GTOM is an extension of the standard topological overlap measure, which traditionally evaluates the extent to which two nodes in a graph share common neighbors [26, 128, 134]. While the conventional Topological Overlap Matrix (TOM) accounts only for direct neighbors, the GTOM generalizes this concept by including indirect neighbors up to a specified path length * d *. In doing so, GTOM captures not only first-order but also higher-order relational similarities within the connectome.

Formally, let * A * denote the binary adjacency matrix of a connectome, and $N_i^{(d)}$ the set of nodes reachable from node * i * within * d * steps. The GTOM between nodes * i * and * j * is defined as:

$$ \text {GTOM}^{(d)}(i, j) = \frac{|N_i^{(d)} \cap N_j^{(d)}| + A_{ij}}{\min \left( |N_i^{(d)}|, |N_j^{(d)}|\right) + 1 - A_{ij}} $$

This normalized metric yields values in the range *[0, 1]*, where 1 indicates perfect topological equivalence within the specified order.

## Appendix H Community Detection using the Louvain Approach with Consensus Clustering

Community detection is a central problem in network science, concerned with identifying groups of nodes–called communities or modules–that are more densely connected internally than with the rest of the network [149–151]. In the context of brain networks, such communities often correspond to functionally specialized subsystems or structurally coherent modules. Detecting these communities allows for the reduction of complex connectivity data into interpretable mesoscale structures, facilitating insights into functional segregation and integrative mechanisms of the brain.

Community detection is fundamentally important because it reveals hidden regularities in complex networks that are not evident from local connectivity alone. In neurobiological networks, such community structures often reflect underlying anatomical subdivisions, synchronized functional activity, or common developmental and evolutionary origins. The Louvain algorithm, due to its scalability, efficiency, and ability to detect hierarchical and weighted community structure, is particularly well-suited for the analysis of brain connectivity matrices such as those derived from GTOM or structural connectomes.

One of the most widely used methods for community detection is the Louvain algorithm, a greedy optimization technique designed to uncover hierarchical modular structures in large networks. The algorithm operates by maximizing the modularity function * Q *, which quantifies the density of edges within communities relative to a random graph null model. The modularity is defined as:

$$ Q = \frac{1}{2m} \sum _{i,j} \left[ A_{ij} - \frac{k_i k_j}{2m} \right] \delta (c_i, c_j), $$

where $A_{ij}$ is the weight of the edge between nodes * i * and * j *, $k_i = \sum _j A_{ij}$ is the strength (weighted degree) of node * i *, $m = \frac{1}{2} \sum _{i,j} A_{ij}$ is the total edge weight in the network, and $\delta (c_i, c_j)$ is the Kronecker delta function which equals 1 if nodes * i * and * j * are assigned to the same community and 0 otherwise. The goal of the Louvain algorithm is to find a partition of the nodes that maximizes * Q *, indicating a configuration where intra-community connections are significantly denser than inter-community connections.

The Louvain method proceeds iteratively in two main phases. In the first phase, each node is initially assigned to its own community. The algorithm then evaluates the change in modularity * Δ Q * for moving a node to the community of each of its neighbors and performs the move that results in the highest modularity gain. This process is repeated for all nodes until no further improvement is possible. In the second phase, a new network is constructed where each community becomes a single meta-node, and edge weights between communities are aggregated. These two steps are repeated recursively on the meta-network until modularity can no longer be increased.

Consensus clustering within the Louvain community detection framework offers several key advantages [151–154]. The Louvain algorithm is inherently stochastic, often producing different community partitions across runs due to random initialization and tie-breaking. Consensus clustering aggregates the results of multiple Louvain runs to produce a partition that is much more stable and reproducible, reducing the influence of randomness on the final community structure . By combining multiple partitions, consensus clustering can enhance the accuracy of community detection. It does this by identifying communities that consistently appear across different runs, thus filtering out spurious or unstable assignments that may arise in any single execution. The approach mitigates the noise and variability introduced by the stochastic nature of Louvain and similar algorithms, leading to more reliable detection of true underlying community structure. Consensus clustering can reveal hierarchical or multi-scale community structures that might be missed in a single run, and can also help in identifying overlapping or fuzzy community boundaries In summary, consensus clustering makes Louvain-based community detection results more stable, accurate, and robust, especially in large, complex, or noisy networks such as those encountered in brain connectomics

## Appendix I Vulnerability Analysis

Let *G = (V, E)* be an undirected, weighted graph representing a brain network, where *V* is the set of nodes (brain regions) and *E* the set of edges (connections). Let $S: E \rightarrow \mathbb {R}$ be a precomputed **significance function**, assigning to each edge *e ∈ E* a real-valued measure of importance (e.g., derived from functional relevance, consistency, or statistical properties) [26, 155].

We define a scalar-valued network metric *f*(*G*), such as the average clustering coefficient, global efficiency, or modularity.

The goal of vulnerability analysis is to evaluate how the network metric *f*(*G*) changes as edges are progressively removed according to their significance. Two types of edge-removal strategies are considered [135]:

- **High-to-low significance removal** (targeting the most important edges first)
- **Low-to-high significance removal** (removing the least important edges first)

Let $E_k^{\uparrow }$ denote the set of the *k* most significant edges (i.e., those with highest values of *S*), and $E_k^{\downarrow }$ the *k* least significant ones. Define the partially pruned graphs:

$$ G_k^{\uparrow } = (V, E \setminus E_k^{\uparrow }), \quad G_k^{\downarrow } = (V, E \setminus E_k^{\downarrow }) $$

Then the **vulnerability curves** are defined as:

$$ V^{\uparrow }(k) = f(G_k^{\uparrow }), \quad V^{\downarrow }(k) = f(G_k^{\downarrow }) $$

where *k = 0, 1, … , |E|*. These curves capture the degradation of the network property *f* as edges are removed.

To assess the non-randomness of this vulnerability profile, the same procedure is repeated on a set of surrogate networks $\{G^{(i)}_{\text {rand}}\}_{i=1}^N$ (e.g., generated via degree-preserving rewiring). This yields corresponding curves:

$$ V^{\uparrow }_{\text {rand}}(k), \quad V^{\downarrow }_{\text {rand}}(k) $$

from which confidence intervals or null distributions can be estimated.

A network is considered **vulnerable** if $V^{\uparrow }(k)$ decays rapidly or differs significantly from the surrogate-based expectations. In contrast, **robustness** implies that *f*(*G*) remains relatively stable across edge removals [156].

## Code availability

https://neuroviisas.med.uni-rostock.de/neuroviisas.shtml

## Declarations

### Code availability

https://neuroviisas.med.uni-rostock.de/neuroviisas.shtml
